# Reversible association with motor proteins (RAMP): A streptavidin-based method to manipulate organelle positioning

**DOI:** 10.1371/journal.pbio.3000279

**Published:** 2019-05-17

**Authors:** Carlos M. Guardia, Raffaella De Pace, Aritra Sen, Amra Saric, Michal Jarnik, David A. Kolin, Ambarish Kunwar, Juan S. Bonifacino

**Affiliations:** 1 Cell Biology and Neurobiology Branch, Eunice Kennedy Shriver National Institute of Child Health and Human Development, National Institutes of Health, Bethesda, Maryland, United States of America; 2 Department of Biosciences and Bioengineering, Indian Institute of Technology Bombay, Powai, Mumbai, Maharashtra, India; UT Southwestern Medical Center, UNITED STATES

## Abstract

We report the development and characterization of a method, named reversible association with motor proteins (RAMP), for manipulation of organelle positioning within the cytoplasm. RAMP consists of coexpressing in cultured cells (i) an organellar protein fused to the streptavidin-binding peptide (SBP) and (ii) motor, neck, and coiled-coil domains from a plus-end–directed or minus-end–directed kinesin fused to streptavidin. The SBP–streptavidin interaction drives accumulation of organelles at the plus or minus end of microtubules, respectively. Importantly, competition of the streptavidin–SBP interaction by the addition of biotin to the culture medium rapidly dissociates the motor construct from the organelle, allowing restoration of normal patterns of organelle transport and distribution. A distinctive feature of this method is that organelles initially accumulate at either end of the microtubule network in the initial state and are subsequently released from this accumulation, allowing analyses of the movement of a synchronized population of organelles by endogenous motors.

## Abstract 2

Reversibly coupling intracellular organelles to microtubule motors, using streptavidin-biotin chemistry, enables the analysis of the relationship between organelle positioning and function.

## Author summary

Intracellular organelles are dynamically distributed throughout the cytoplasm by virtue of regulated interactions with microtubule motors. A significant development over the past few years has been the realization that the spatial distribution of organelles influences their functions. However, in most cases, the exact relationship between organelle dynamics and function is not well-understood. Investigating this relationship requires methods to experimentally manipulate organelle dynamics. In this article, we describe a novel, to our knowledge, method to reposition organelles within the cytoplasm by artificial coupling to microtubule motor domains. This method, named reversible association with motor proteins (RAMP), consists of coexpressing an organellar protein appended with a streptavidin-binding peptide and an anterograde or retrograde motor domain fused to streptavidin. Interaction of the streptavidin-binding peptide with streptavidin results in coupling of the organelle to the motor, leading to redistribution of the organelle to the periphery or the center of the cell, respectively. Importantly, this redistribution can be reversed by the addition of the vitamin biotin, which competes off the streptavidin-binding peptide–streptavidin interaction. We show that this method is applicable to various organelles, including lysosomes, mitochondria, endoplasmic reticulum, and peroxisomes, enabling future studies of the properties of these organelles at different cellular locations.

## Introduction

A hallmark of eukaryotic cells is the spatial organization of their cytoplasm into an array of membrane-bounded organelles. This organization is highly plastic because the cytoplasmic distribution of organelles varies depending on the cell type, differentiation state, environmental cues, stress conditions, and other physiological and pathological factors [1,2]. The positioning of organelles often influences their activities, allowing for local control of organellar functions. Changes in organelle positioning depend on the ability of the individual organelles to move throughout the cytoplasm. Long-range, bidirectional movement occurs on microtubule tracks and is driven by attachment of the organelles to kinesin and dynein motors. Of the approximately 45 kinesins encoded in mammalian genomes, most drive cargo movement from the minus end to the plus end of microtubules (i.e., anterograde transport), whereas only a few move cargo in the opposite direction (i.e., retrograde transport) [3]. Coupling of different organelles to kinesins depends on an assortment of adaptor or scaffold proteins, as well as various regulators. In contrast to the multiplicity of kinesins, in mammals, there is only one cytoplasmic dynein, which mediates nearly all cargo transport in the plus-to-minus–end direction [4,5]. Attachment of different organelles to the single dynein molecule is achieved through the use of multiple dynein subunit isoforms, cargo-specific adaptors, and regulators [6].

The ability of microtubule motors to move organelles can be experimentally harnessed to alter organelle positioning and thus assess the influence of positioning on function [1,2]. Methods to manipulate organelle positioning can be additionally used to identify motors, adaptors, and regulators that mediate movement of specific organelles on different microtubule tracks or to test for protein colocalization to the same organelle [7–12]. Previous studies used inducible heterodimerization modules to force artificial coupling of a motor protein or domain to an organellar protein or cargo adaptor. Some of these modules undergo dimerization upon addition of a ligand, as is the case for FK506-binding protein (FKBP)–FKBP-rapamycin binding domain (FRB) and rapalog [7–9,12], gibberellin insensitive dwarf1 (GD1)–gibberellin insensitive (GAI) and gibberellin [13], and SNAPtag–HaloTag and CoreM [14]. Other modules dimerize upon exposure to light, as is the case for the cryptochrome 2 (CRY2)–cryptochrome-interacting basic helix–loop–helix (CIB1) [15], light–oxygen–voltage–peptide domain (LOVpep)–engineered postsynaptic density protein (PSD95), *Drosophila* disc large tumor suppressor (DLG1), and zonula occludens-1 protein (ZO-1) (PDZ) domain (ePDZ) [11,16], and UV resistance locus 8 (UVR8)–constitutively photomorphogenic 1 (COP1) [17] pairs. Each of these systems has advantages and disadvantages concerning their speed of dimerization, reversibility, ease of use, requirement of special equipment, adaptability to both imaging and biochemical analyses, and potential for high-throughput applications. Thus, the choice of a particular system depends on the biological problem under study, as well as the access to appropriate reagents and equipment. Moreover, it is often necessary to test more than one system, alone or in combination, to find the most suitable approach for specific applications. In this context, it is desirable to have a wide selection of dimerization methods with different properties.

**Fig 1:**
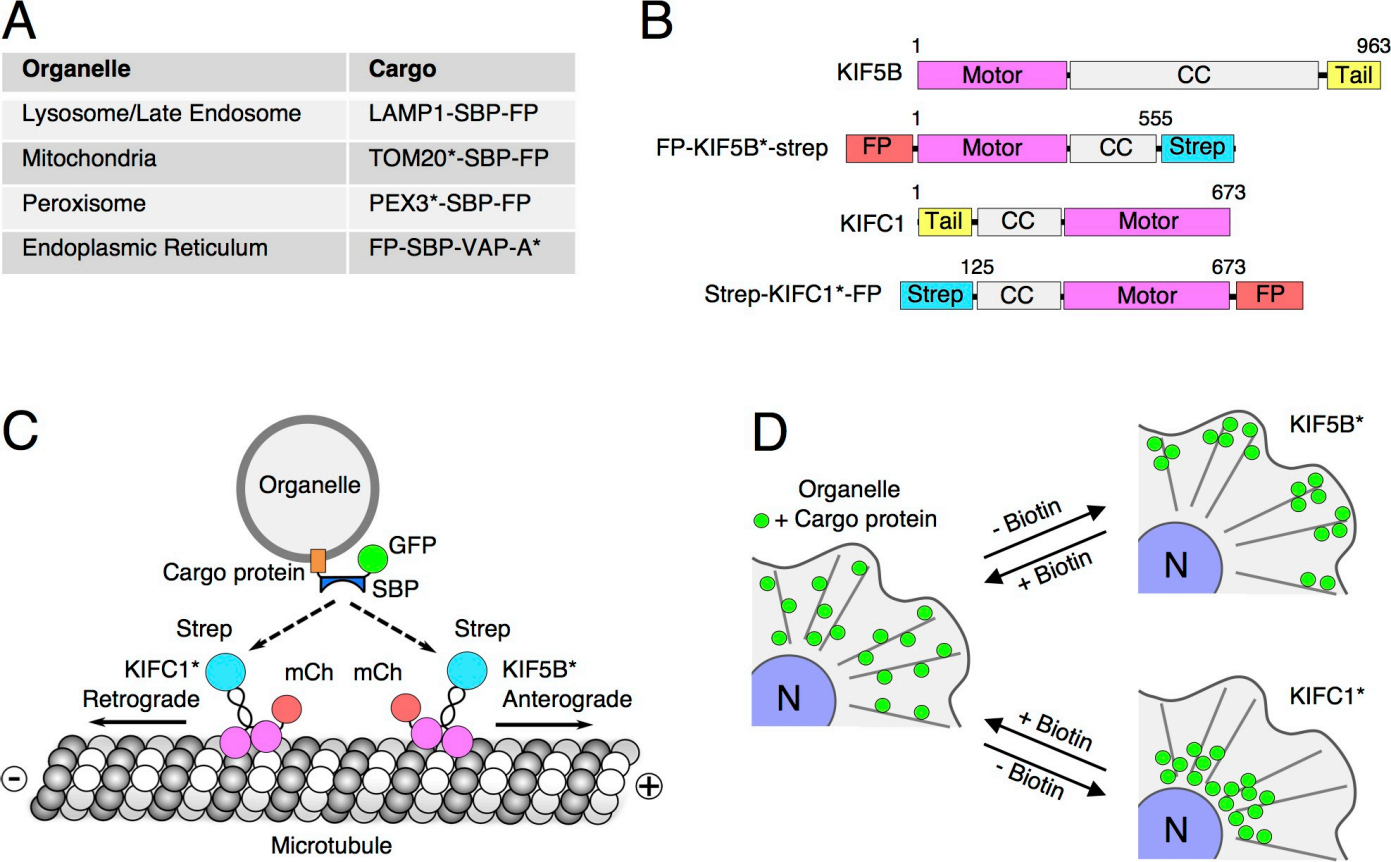
Schematics of RAMP. (A) RAMP cargo constructs used in this study. (B) RAMP motor constructs used in this study. Amino-acid numbers are indicated. Tail is the natural cargo-binding domain. In some experiments, the FP was replaced by a HA epitope. Additional motor constructs are shown in S8B Fig. The asterisks indicate that only fragments of the cargo and motor proteins were used to make the constructs (for more details, see Methods section). (C) Coexpression of the RAMP cargo and motor constructs results in coupling of the organelle to the motor, with consequent motor activation and organelle redistribution in anterograde (KIF5B*-based construct) or retrograde (KIFC1*-based construct) direction. (D) Representation of the redistribution of organelles upon expression of RAMP constructs. Addition of biotin outcompetes the SBP–streptavidin interaction, reversing the peripheral or perinuclear accumulation of the organelle. CC, coiled coil; FP, fluorescent protein; GFP, green fluorescent protein; HA, hemagglutinin; KIF, kinesin superfamily; LAMP, lysosome-associated membrane protein; mCh, mCherry; PEX, peroxin; RAMP, reversible association with motor proteins; SBP, streptavidin-binding protein; Strep, streptavidin; TOM, translocase of the outer membrane; VAP, VAMP-associated protein.

Here, we describe an approach for coupling microtubule motors to organelles that relies on the interaction of streptavidin with the streptavidin-binding peptide (SBP), and its reversal by the addition of D-biotin. These interaction partners have been previously used for synchronization of protein transport along the secretory pathway (known as the “RUSH” method, for retention using selective hooks) [18] and for the control of protein trafficking by reversible masking of sorting signals (“CUTE” method, for controlled unmasking of targeting elements) [19]. Our method, named “reversible association with motor proteins” (RAMP), consists of coexpressing in cultured cells (i) an organellar protein fused to SBP and a fluorescent protein (i.e., RAMP cargo) (Fig 1A) and (ii) motor and coiled-coil domains of a plus-end–directed (KIF5B) or minus-end–directed (KIFC1) kinesin fused to streptavidin and another fluorescent protein (i.e., RAMP motor) (Fig 1B). The SBP–streptavidin interaction (Fig 1C) drives accumulation of organelles at the plus or minus end of microtubules, respectively (Fig 1D). Most importantly, competition of the streptavidin–SBP interaction by the addition of biotin to the culture medium rapidly dissociates the motor construct from the organelle, allowing restoration of normal patterns of organelle transport and distribution (Fig 1D). This approach differs from previous methods for manipulating organelle positioning and motility in that proteins accumulate at the ends of microtubules in the initial state but return to their normal distributions upon addition of biotin. Thus, in addition to its rapid and complete reversibility, the main advantage of this approach is the ability to synchronize the movement of a whole population of organelles from either end of the microtubule network.

## Results

### Development and validation of RAMP

For use in the RAMP system, we constructed plasmids encoding full-length forms or part of organellar proteins such as the lysosomal/late-endosomal lysosome-associated membrane protein 1 (LAMP1), mitochondrial translocase of the outer membrane 20 (TOM20), peroxisomal peroxin 3 (PEX3), or endoplasmic reticulum (ER) VAMP-associated protein (VAP)-A, appended at their cytosolically exposed N- or C-termini with the 38-amino–acid SBP and a fluorescent protein (green fluorescent protein [GFP] or mCherry) (Fig 1A). These plasmids were co-transfected with plasmids encoding the motor, neck, and part of the coiled-coil domains from the plus-end–directed kinesin-1 KIF5B (denoted KIF5B*) [20] or minus-end–directed kinesin-14 KIFC1 (denoted KIFC1*) [21], both fused to streptavidin (strep) and another fluorescent protein (mCherry or GFP) (Fig 1B). These kinesin constructs lacked their cargo-recognition tail domains, so they could only bind to organelles via the SBP–streptavidin interaction (Fig 1C). Different combinations of organellar and motor constructs were expressed by transient co-transfection into cultured cells, resulting in accumulation of organelles at the plus or minus ends of microtubules, depending on the motor construct used (Fig 1D). This accumulation could be reversed by the addition of biotin (Fig 1D).

**Fig 2:**
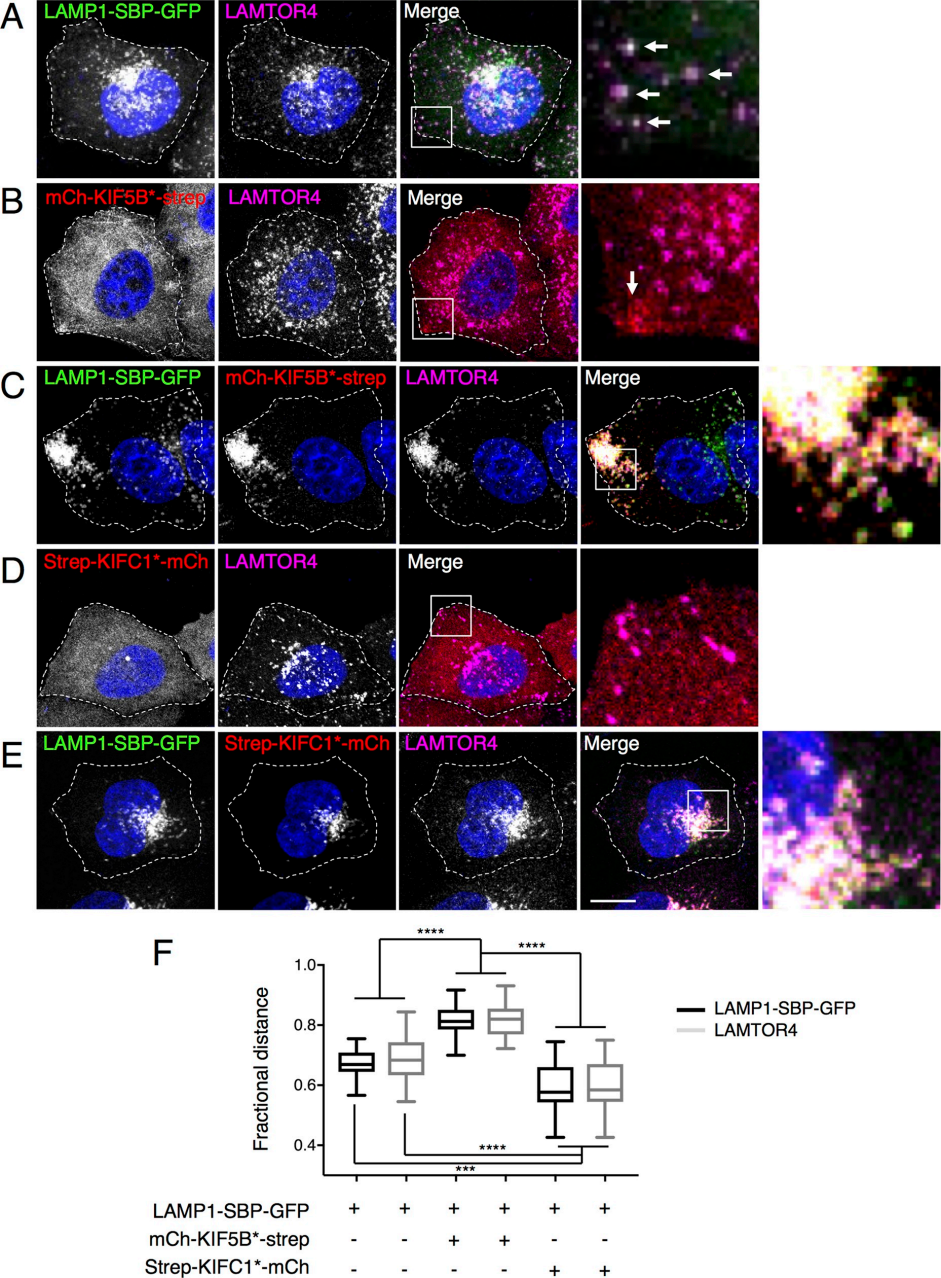
Redistribution of lysosomes by expression of RAMP constructs. (A) Colocalization of LAMP1-SBP-GFP with endogenous LAMTOR4 in HeLa cells. Arrows indicate colocalization. (B) mCh-KIF5B*-strep is largely cytosolic and does not disrupt the distribution of lysosomes stained for endogenous LAMTOR4. Arrow shows localization of a small amount of mCh-KIF5B*-strep in a cell protrusion. (C) Coexpressed LAMP1-SBP-GFP and mCh-KIF5B*-strep accumulate on LAMTOR4-stained lysosomes at peripheral cell protrusions. (D) Strep-KIFC1*-mCh is cytosolic and does not disrupt the distribution of lysosomes stained for LAMTOR4. (E) Coexpressed LAMP1-SBP-GFP and strep-KIFC1*-mCh accumulate on LAMTOR4-stained lysosomes in the perinuclear area of the cell. Nuclei were stained with DAPI. Cell edges are outlined. Scale bar: 10 μm. Rightmost panels are 4.5× magnifications of the boxed areas in the merged images in each row. (F) Box-and-whisker plots represent the fractional distance distribution (*f* = 95%) of lysosomes in the conditions from panels A, C, and E (see S4 Fig and Methods section for details). Summary data available as Supporting Information (S1_Data.xlsx). GFP, green fluorescent protein; KIF, kinesin superfamily; LAMP, lysosome-associated membrane protein; LAMTOR4, late endosomal/lysosomal adaptor and MAPK and MTOR activator 4; mCh, mCherry; RAMP, reversible association with motor proteins; SBP, streptavidin-binding protein; strep, streptavidin.

Initial tests of the RAMP system were performed for lysosomes in HeLa cells (Fig 2 and S1–S3 Figs). In these cells, lysosomes are distributed throughout the cytoplasm, although with a higher concentration in the perinuclear area of the cell. This distribution is dynamic, as lysosomes can move bidirectionally along microtubules radiating from the microtubule-organizing center (MTOC) [22,23] (S1A Fig). Plus-end–directed (anterograde) and minus-end–directed (retrograde) movement of lysosomes is driven by kinesin and dynein–dynactin motors, respectively [2,24]. We observed that singly expressed LAMP1-SBP-GFP localized to lysosomes, as detected by costaining for the endogenous lysosomal marker late endosomal/lysosomal adaptor and MAPK and MTOR activator 4 (LAMTOR4) [25] (Fig 2A and 2F). Singly expressed mCh-KIF5B*-strep, on the other hand, exhibited a largely cytosolic localization, albeit with a slight accumulation at cell protrusions, where the plus ends of microtubule bundles are more abundant (Fig 2B, arrow). Expression of LAMP1-SBP-GFP or mCh-KIF5B*-strep alone did not affect the distribution of lysosomes (Fig 2A and 2B) or the structure of the microtubule network (S1B and S1C Fig). When LAMP1-SBP-GFP and mCh-KIF5B*-strep were expressed together, however, we observed a dramatic redistribution of both proteins, as well as lysosomes labeled for LAMTOR4, to the cell periphery, particularly at cell protrusions (Fig 2C and 2F). This perturbation did not affect the integrity of the microtubule network (S1D Fig).

Singly expressed strep-KIFC1*-mCh was also largely cytosolic and did not affect the distribution of lysosomes (Fig 2D) or microtubules (S1E Fig). However, upon coexpression with LAMP1-SBP-GFP, both constructs colocalized to lysosomes clustered in the pericentrosomal area of the cells (Fig 2E and 2F) without perturbing the microtubules (S1F Fig). Similar results were obtained upon coexpression of LAMP1-SBP-GFP with either mCh-KIF5B*-strep or strep-KIFC1*-mCh in other nonpolarized cell lines such as COS-7 and HT-1080 (S2A and S2B Fig).

The above results demonstrated that the coupling of lysosomes to KIF5B* or KIFC1* via the SBP–streptavidin interaction was sufficient to reconstitute active kinesin motors and thus redistribute lysosomes to the periphery or the center of the cell, respectively.

Control experiments showed that endosomes containing transferrin receptor (TfR) remained distinct and were not redistributed under these conditions (S2C and S2D Fig), indicating that the effects of RAMP were specific for the target organelle. In addition, we observed that both the peripherally and centrally clustered LAMP1-SBP-GFP–positive lysosomes in HeLa cells accumulated the fluorescent acidotropic probe LysoTracker Blue (S3A and S3D Fig) and fluid-phase endocytic marker Alexa Fluor 647-dextran (AF647-dextran) (S3B and S3E Fig) and hydrolyzed the fluorogenic protease substrate dye-quenched bovine serum albumin (DQ-BSA) (S3C and S3F Fig). Thus, the redistribution of lysosomes induced by RAMP was specific and did not grossly alter their functional properties.

**Fig 3:**
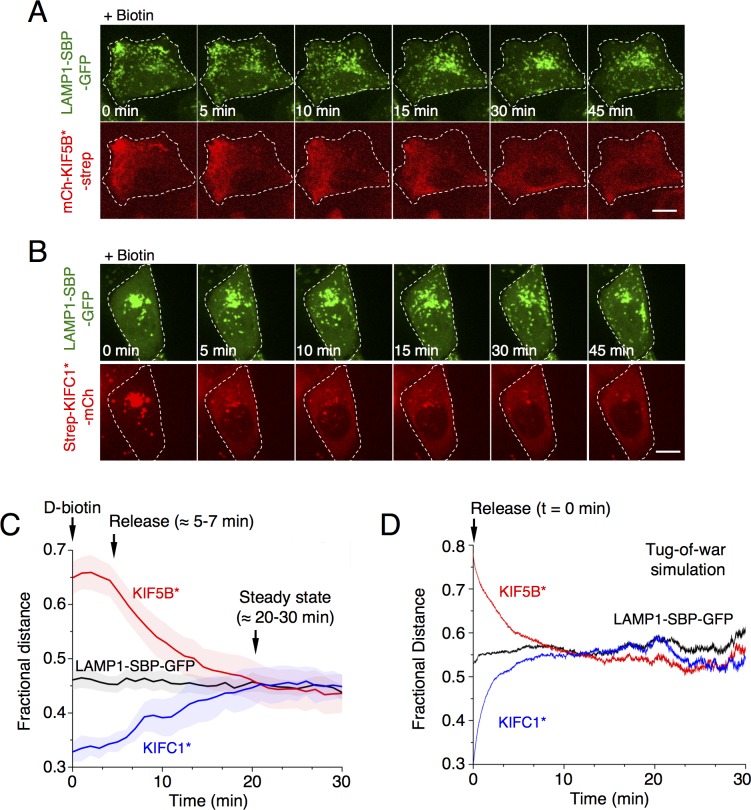
Analysis of the reversal of lysosome redistribution after addition of biotin. (A,B) HeLa cells were co-transfected with plasmids encoding (A) LAMP1-SBP-GFP and mCh-KIF5B*-strep (see S1 Movie) and (B) LAMP1-SBP-GFP and strep-KIFC1*-mCh (see S2 Movie), resulting in the accumulation of lysosomes at the periphery or center of the cells, respectively. At 24 h after transfection, biotin was added, and the return of lysosomes to their normal distribution was recorded by live-cell imaging. Scale bars: 10 μm. (C) Quantification of the reversal of lysosome redistribution from S1 and S2 Movies (average fractional distance (*f* = 75%) of lysosomes as a function of time after biotin addition), in comparison to lysosomes expressing only LAMP-SBP-GFP (also check S4 Fig and Methods for details). Values are the mean ± SD from three independent experiments. (D) Results from simulations of a computational model based on a stochastic model of tug-of-war (S1 Text) show the exponential recovery, reaching normal distribution in a WT context of lysosomes undergoing tug-of-war with the endogenous KIF5B and KIF1B kinesins and dynein [28]. Values are the mean ± SEM from 500 independent cargo initial configurations for each condition. Summary data available as Supporting Information (S1_Data.xlsx). GFP, green fluorescent protein; KIF, kinesin superfamily; LAMP, lysosome-associated membrane protein; mCh, mCherry; SBP, streptavidin-binding protein; strep, streptavidin; WT, wild type.

Addition of 50 μM biotin to cells coexpressing LAMP1-SBP-GFP and mCh-KIF5B*-strep rapidly dissociated mCh-KIF5B*-strep from the lysosomes and reversed the accumulation of both proteins at peripheral sites (Fig 3A and S1 Movie). Similarly, biotin addition dissociated strep-KIFC1*-mCh from the centrally clustered lysosomes, allowing lysosome redistribution towards the cell periphery (Fig 3B and S2 Movie). Quantitative image analysis (S4 Fig) showed that, in both cases, lysosomes recovered their normal steady-state distribution approximately 20–30 minutes after the addition of biotin (Fig 3C). This recovery rate was slower than that predicted by computational methods used to study bidirectional cargo transport undergoing tug-of-war between motor molecules (Fig 3D, and snapshots of the simulations shown in S6A and S6B Fig) (see also S1 Text) [26,27]. The discrepancy between in vivo and in silico experiments could be explained by the contribution of biotin uptake and SBP–streptavidin dissociation rates to the overall kinetics of recovery. Taken together, all of the above results demonstrated the validity of RAMP as a method to redistribute lysosomes in a reversible manner and provided a quantitative framework to analyze the experimental data.

### A previously used BicD2 fragment has a dominant-negative effect

Previous methods to redistribute organelles towards microtubule minus ends used fusions to an N-terminal fragment from the dynein–dynactin cargo adaptor bicaudal D homolog 2 (BicD2) [9,11,29,30]. This fragment (herein denoted BicD2*) comprises the region that interacts with dynein and dynactin but lacks the C-terminal cargo-binding domain [31]. We observed that an mCh-BicD2*-strep fusion protein (S5A Fig) indeed caused clustering of LAMP1-SBP-GFP-containing lysosomes at the cell center (S5B Fig). Furthermore, upon addition of biotin, lysosomes were released from their central location (S5B Fig and S3 Movie). However, we noticed that rather than returning to their normal steady-state distribution, the released lysosomes became clustered at cell protrusions (S5B Fig and S3 Movie). Expression of mCh-BicD2*-strep alone also caused peripheral accumulation of lysosomes labeled for endogenous LAMP1 and dispersal of endosomes labeled for TfR (S5C and S5D Fig). These experiments thus demonstrated that the N-terminal fragment of BicD2 behaves as a dominant-negative mutant that causes lysosome dispersal per se, probably by outcompeting an endogenous adaptor of lysosomes to dynein–dynactin such as Rab-interacting lysosomal protein (RILP) [32], JNK-interacting protein 3 (JIP3) [33,34], or JIP4 [35]. For this reason, our experiments to cluster organelles at the cell center were done using constructs based on KIFC1* rather than BicD2*.

### Analysis of axonal and dendritic transport of lysosomes using RAMP

Next, we extended the testing of RAMP to lysosomes in neurons. Unlike HeLa cells, neurons are highly polarized cells that have specialized peripheral domains such as axons and dendrites with distinct compositions of cytoskeletal structures and organelles [36–38]. The microtubule cytoskeleton, in particular, exhibits a remarkably polarized organization. In the axon, microtubules are long and unidirectional, with their plus ends pointing towards the axon terminal [39,40]. In contrast, dendritic microtubules are short and have a mixed orientation [39–41]. Axonal and dendritic microtubules also have different patterns of tubulin post-translational modifications and microtubule-associated proteins (MAPs) [42,43]. The distinct properties of axonal and dendritic microtubules allow for specialized forms of organelle movement that are not present in other cells. For example, KIF5/kinesin-1 members predominantly move cargo towards axon terminals, while KIF1/kinesin-3 members can move cargo in different neuronal domains [44–47]. How the neuron acquires and maintains this highly polarized organization of microtubules and organelles is a topic of intense interest in neurobiology. The development of RAMP provides a new, to our knowledge, tool to unravel the mechanisms that underlie this organization.

**Fig 4:**
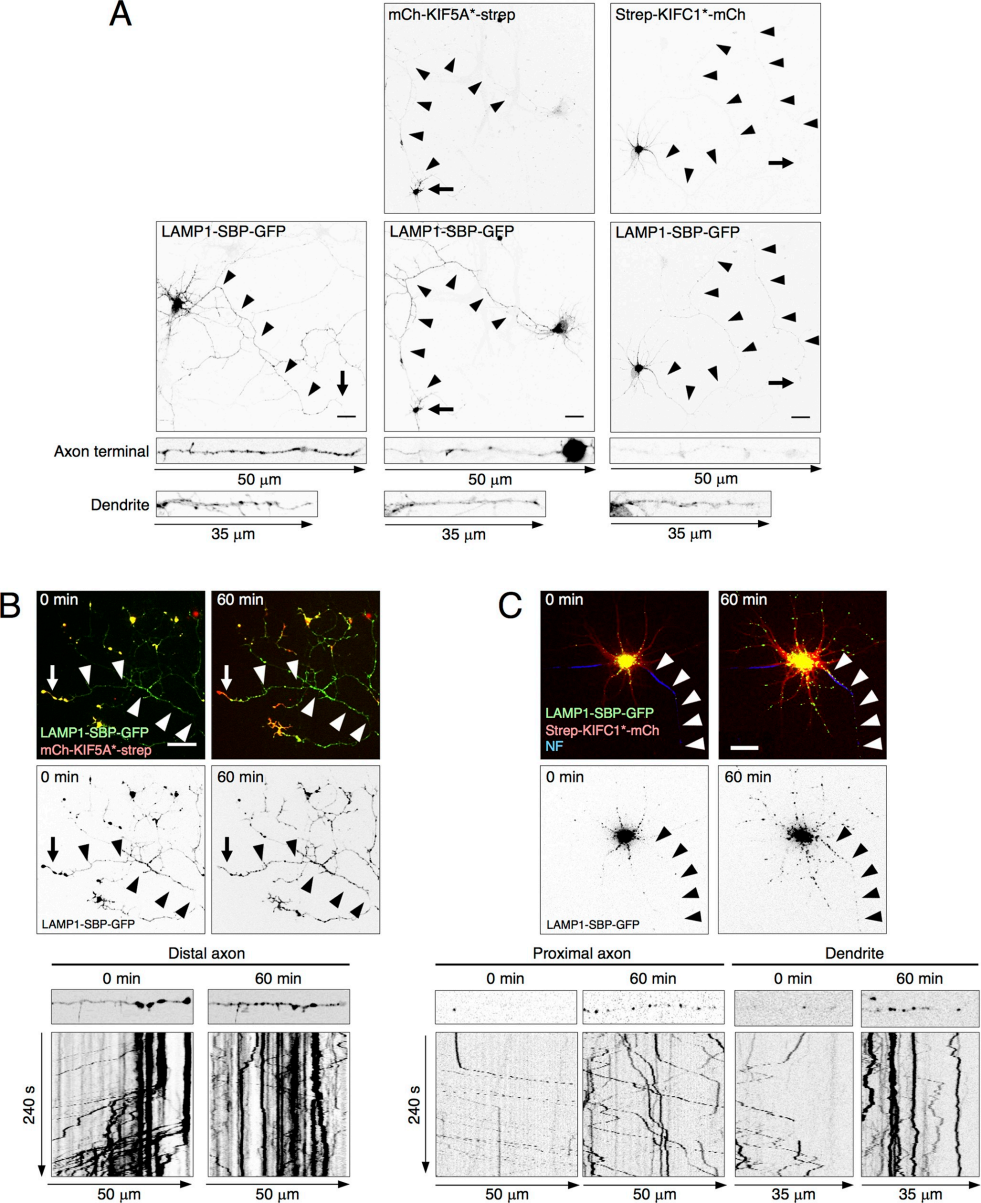
Analysis of polarized transport of neuronal lysosomes using RAMP. (A) DIV5 rat hippocampal neurons were transfected with plasmids encoding LAMP1-SBP-GFP alone (first column) or together with plasmids encoding mCh-KIF5A*-strep (second column) or strep-KIFC1*-mCh (third column). Cells were cultured in the presence of NeutrAvidin to remove biotin from the medium. The following day, neurons were fixed and imaged for GFP and mCherry fluorescence. The top two rows show single neurons in the absence of biotin. The bottom strips show 50 μm of straightened axon terminal and 35 μm of the dendrite portion from each of the above neurons. (B,C) DIV5 neurons were transfected with plasmids encoding LAMP1-SBP-GFP and mCh-KIF5A*-strep (B) or strep-KIFC1*-mCh (C), leading to the accumulation of lysosomes at axon tips and soma, respectively. The following day, live neurons were stained with a CF640R-conjugated antibody to neurofascin to label the AIS and thus identify the axon. Biotin was added, and neurons were then imaged live for all fluorescent probes. The top two rows show frames at 0 and 60 minutes after the addition of biotin. The bottom panels show 50 μm of straightened portions and kymographs of the distal axon (B), proximal axon (C), or dendrites (C), as indicated, beginning at 0 and 60 minutes after the addition of biotin. In the kymographs, positive slopes represent retrograde transport, negative slopes anterograde transport, and vertical lines stationary foci. Arrowheads mark the trajectory of the axon. Arrows indicate the axon tip. Scale bars: 20 μm. Summary data available as Supporting Information (S1_Data.xlsx). AIS, axon initial segment; DIV5, day in vitro 5; GFP, green fluorescent protein; KIF, kinesin superfamily; LAMP, lysosome-associated membrane protein; mCh, mCherry; RAMP, reversible association with motor proteins; SBP, streptavidin-binding protein; strep, streptavidin.

To examine the characteristics of lysosome transport in the axon and dendrites, we started by coexpressing LAMP1-SBP-GFP and mCh-KIF5A*-strep in rat hippocampal neurons in primary culture. In these experiments, we used a motor construct based on KIF5A because this KIF5 paralog is specifically expressed in neurons [48]. We observed that coexpression of LAMP1-SBP-GFP and mCh-KIF5A*-strep resulted in accumulation of lysosomes at the tips of axons but not dendrites (Fig 4A, axon tips indicated by arrows), in accordance with the preference of KIF5A for axonal microtubules [29,44,45,49]. Similar results were obtained using a motor construct based on the ubiquitously expressed KIF5B (S7A Fig). Upon addition of biotin, lysosomes that had accumulated at the axon tip by the action of mCh-KIF5A*-strep were rapidly mobilized in the retrograde direction (lines with positive slopes in the kymographs), with an initial rate of 0.6 μm/s (Fig 4B and S7B Fig).

We also examined the effect of coexpressing LAMP1-SBP-GFP with mCh-KIFC1*-strep. We observed that this manipulation resulted in accumulation of lysosomes in the soma, with concomitant depletion of lysosomes from the axon and reduction in the number of lysosomes in the dendrites (Fig 4A, right panels). Nevertheless, the remaining dendritic lysosomes had a dotted distribution along the entire dendrite (Fig 4A). These findings demonstrated that the KIFC1* motor can drive movement in both axon and dendrites but with different end results, consistent with the distinct organization of axonal and dendritic microtubules. Also, in this case, treatment with biotin rapidly restored lysosome transport in both the axon (initial average rate: 1.1 μm/s) and the dendrites (initial average rates: 0.6 μm/s anterograde, 0.7 μm/s retrograde) (Fig 4C and S7B Fig). Altogether, these studies demonstrated the suitability of RAMP for analyzing the polarized transport of lysosomes in different neuronal domains.

### Shape changes of mitochondria accumulated in the cell periphery by RAMP

**Fig 5:**
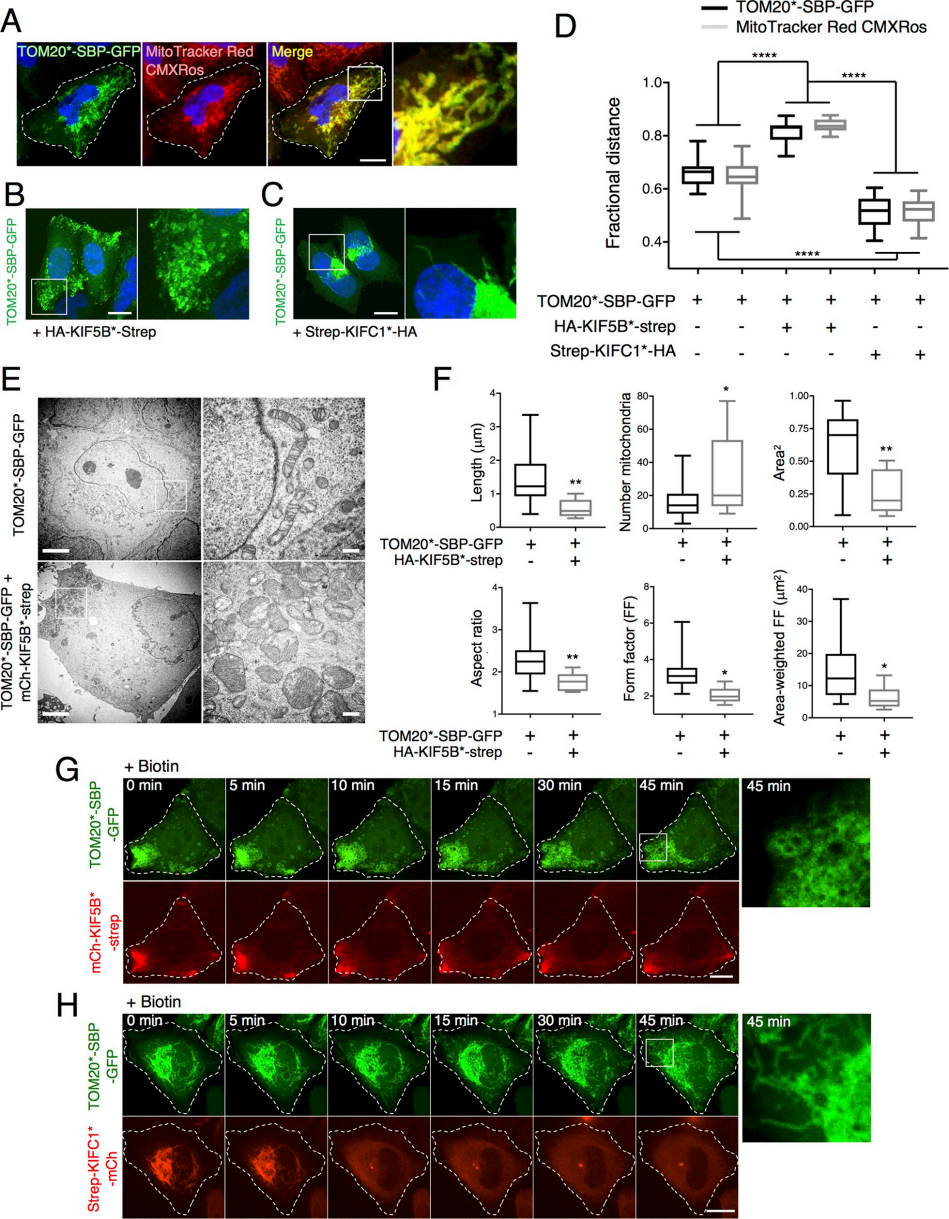
Redistribution of mitochondria to the cell periphery alters their shape. (A) Colocalization of TOM20*-SBP-GFP with 25 nM MitoTracker Red CMXRos in HeLa cells incubated for 30 minutes at 37°C, 5% CO_2_. (B) Coexpression of TOM20*-SBP-GFP with HA-KIF5B*-strep causes peripheral redistribution of mitochondria and alters mitochondrial shape. (C) Coexpression of TOM20*-SBP-GFP with strep-KIFC1*-mCh causes central redistribution of mitochondria without affecting their morphology. In A–C, nuclei were stained with DAPI. The rightmost images in A–C are 3.5× magnified views of the boxed areas. Scale bars: 10 μm. (D) Box-and-whisker plots representing the fractional distance distribution (*f* = 95%) of mitochondria in the conditions from panels A, B, and C (see S4 Fig and Methods section for details). Summary data available as Supporting Information (S1_Data.xlsx). (E) Electron microscopy of cells expressing TOM20*-SBP-GFP alone (top row) or with HA-KIF5B*-strep (bottom row), where rounder mitochondria with normal internal organization is shown. Images on the right are magnifications of boxed areas on the left. Left column scale bar: 4 μm; right column scale bar: 0.6 μm. (F) Quantification of the phenotype observed in (B), using mitochondrial shape descriptors [50] (see Methods section for more details). Summary data available as Supporting Information (S1_Data.xlsx). (G,H) HeLa cells coexpressing TOM20*-SBP-GFP with mCh-KIF5B*-strep (G) (S4 Movie) or strep-KIFC1*-mCh (H) (S5 Movie) were incubated with biotin and analyzed by live-cell imaging. Scale bar: 10 μm. Rightmost images are 4.5× enlargements of the boxed areas. GFP, green fluorescent protein; HA, hemagglutinin; KIF, kinesin superfamily; mCh, mCherry; SBP, streptavidin-binding protein; strep, streptavidin; TOM, translocase of the outer membrane.

To test the applicability of RAMP to other organelles, we extended our analyses to mitochondria in HeLa cells (Fig 5). Coexpression of TOM20*-SBP-GFP (Fig 5A) with mCh-KIF5B*-strep or strep-KIFC1*-mCh also caused accumulation of mitochondria at the periphery or the center of the cell, respectively (Fig 5B and 5C; see Fig 5D for quantification). Interestingly, both fluorescence (Fig 5B) and electron microscopy (Fig 5E) showed that mitochondria became rounder when accumulated at the cell periphery by mCh-KIF5B*-strep. Quantification of different morphological parameters from the fluorescent images confirmed that the peripherally distributed mitochondria had a rounder shape, as evidenced by their shorter length, as well as lower aspect ratio and form factor (Fig 5F) [50]. In addition, this analysis revealed that peripherally distributed mitochondria were more numerous and had a smaller area (Fig 5F) [50]. Electron microscopy also showed that, despite their altered shape, the peripherally accumulated mitochondria retained their internal structure, including typical cristae (Fig 5E). They also retained a negative membrane potential, as determined by staining with MitoTracker Red CMXRos [51–53] (S8A Fig). Similar changes in mitochondrial morphology were observed upon peripheral redistribution by coexpression of TOM20*-SBP-GFP with three other constructs comprising the coiled-coil and motor domains from the plus-end–directed kinesin-3 members KIF1A and KIF13A and kinesin-2 member KIF17, all appended with mCherry and streptavidin (S8B and S8C Fig). Since other than mediating plus-end–directed transport, KIF1A, KIF13A, and KIF17 are structurally and functionally distinct from KIF5B, these results indicated that the change in mitochondrial morphology is not specific to KIF5B but is likely dependent on the strong anterograde pulling force and relocation to the cell periphery induced by kinesins [54]. Mitochondria that accumulated at the cell periphery by expression of TOM20*-SBP-GFP with mCh-KIF5B*-strep retained their rounder shape even after reversal of their accumulation by the addition of biotin (Fig 5G, S4 Movie). In contrast, accumulation of mitochondria at the cell center by expression of strep-KIFC1*-mCh (Fig 5C) and reversal of this accumulation by addition of biotin (Fig 5H, S5 Movie) had no effect on the overall shape of mitochondria.

Mitochondrial morphology is maintained by a dynamic equilibrium between fusion and fission. Mitochondrial fusion is achieved by mitofusins 1 and 2 [55], two GTPases that tether mitochondria and allow membrane fusion [56,57]. Mitochondrial fission is coordinated by a much more complicated mechanism involving the ER [58], actin polymerization [59], myosin [60], ER–plasma membrane contacts [61], and several dynamin‐like proteins and their adaptors [62–64]. RAMP could thus be used to investigate how mitochondrial positioning affects these machineries.

### Manipulation of the distribution of ER and peroxisomes using RAMP

**Fig 6:**
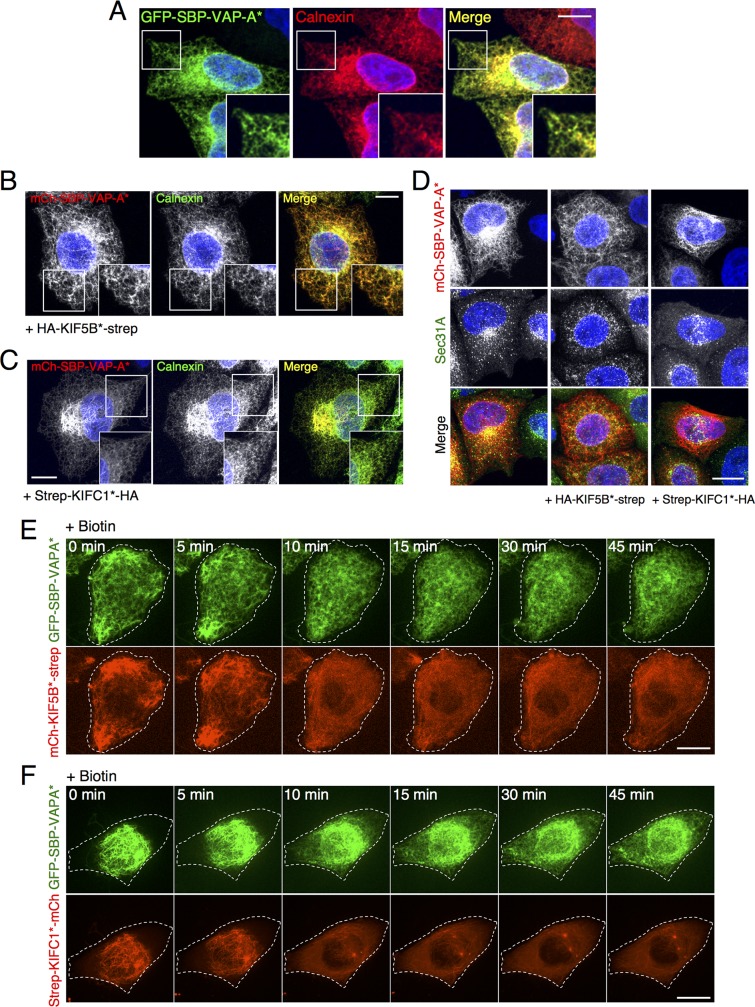
Redistribution of the ER with RAMP. (A) Confocal microscopy of HeLa cells expressing GFP-SBP-VAP-A* shows localization of the fusion protein to a network characteristic of the ER shown by costaining for endogenous calnexin, a luminal ER-resident protein. Insets correspond to 1.6× magnified views of the boxed areas. (B,C) HeLa cells were co-transfected with plasmids encoding mCh-SBP-VAP-A* and HA-KIF5B*-strep (B) or strep-KIFC1*-HA (C) and stained for endogenous calnexin. At 24 h after transfection, cells were rinsed with PBS and immediately fixed with paraformaldehyde. Notice that the ER network mostly follows the redistribution caused by RAMP with mCh-SBP-VAP-A*. Insets correspond to 1.2× magnified views of the boxed areas. (D) HeLa cells were co-transfected with plasmids encoding mCh-SBP-VAP-A* and HA-KIF5B*-strep or strep-KIFC1*-HA and stained for endogenous Sec31A. At 24 h after transfection in absence of biotin, cells were rinsed with PBS and immediately fixed with paraformaldehyde. Notice that the ERESs also follow the redistribution caused by RAMP. In A–D, nuclei were stained with DAPI. (E,F) HeLa cells coexpressing GFP-SBP-VAP-A* and mCh-KIF5B*-strep (E) or strep-KIFC1*-mCh (F) were analyzed by time-lapse microscopy after addition of biotin (see S6 and S7 Movies, respectively). Scale bars: 10 μm. ER, endoplasmic reticulum; ERES, ER exit site; GFP, green fluorescent protein; HA, hemagglutinin; KIF, kinesin superfamily; mCh, mCherry; RAMP, reversible association with motor proteins; SBP, streptavidin-binding protein; Sec31A, secretory protein 31A; strep, streptavidin; VAP, VAMP-associated protein.

We also used RAMP to manipulate the distribution of the ER (Fig 6) and peroxisomes (S9 Fig) in HeLa cells. When GFP-SBP-VAP-A* was expressed alone, it localized to the ER network (Fig 6A), and its coexpression with hemagglutinin (HA)-tagged KIF5B*-strep or strep-KIFC1* shifted its overall distribution towards the cell periphery or the cell center, respectively (Fig 6B and 6C). In all cases, mCh-SBP-VAP-A*–positive structures costained for the endogenous ER protein calnexin, indicating that RAMP caused redistribution of the ER and not just mCh-SBP-VAP-A* (Fig 6B and 6C). The redistribution of the ER to the center or periphery of the cell, however, was partial and did not lead to complete disruption of the ER network. The residual structures could be resistant to redistribution because they are tightly tethered to the nucleus, the plasma membrane, or cytoskeletal structures. ER exit sites (ERESs) [65] also underwent redistribution after RAMP (Fig 6D), demonstrating that ER-associated structures can also be manipulated with this tool. As for other organelles, addition of biotin restored the normal distribution of GFP-SBP-VAP-A* (Fig 6E and 6F; S6 and S7 Movies).

PEX3*-SBP-GFP alone colocalized with endogenous peroxisomal membrane protein 70 (PMP70) but not early endosome antigen 1 (EEA1) (S9A Fig). Coexpression of PEX3*-SBP-GFP with HA-KIF5B*-strep or Strep-KIFC1*-HA caused accumulation of peroxisomes at the cell periphery or the cell center, respectively (S9B and S9C Fig; S9D Fig for a quantification of the redistributions), as previously shown for lysosomes (Figs 2 and 3) and mitochondria (Fig 5). Treatment with biotin dissociated the kinesin constructs and allowed the peroxisomes to return to their normal steady-state distribution (S9E and S9F Fig; S8 and S9 Movies).

## Discussion

We have presented the development and characterization of RAMP as a new method to manipulate the positioning of organelles within cells. RAMP can be applied to various organelles (lysosomes, mitochondria, ER, peroxisomes) and cell types (HeLa, COS-7, HT-1080, hippocampal neurons). It can be used for some of the same purposes as other methods for manipulating organelle positioning. For example, RAMP allows analyses of whether two or more proteins are present in the same organelle, whether a protein localizes to or interacts with a specific organelle, and how organelle repositioning affects cellular functions such as polarized sorting, cell adhesion and migration, metabolism, cell division, etc. However, RAMP has distinctive features that make it a good alternative or complement to other methods for manipulating organelle positioning. An important difference from other methods is that in the initial state, organelles accumulate at the plus or minus end of microtubules by virtue of the SBP–streptavidin interaction, and this accumulation can be reversed by the addition of biotin. These features allow for synchronization of the movement of an organelle cohort from either end of the microtubule network, simplifying analyses of the kinetics of organelle transport by endogenous motors as well as the involvement of specific proteins in this movement. It is important to note that this method is reversible insofar as the normal distribution of an organelle can be restored by the addition of biotin. The extremely high affinity of biotin for streptavidin, however, prevents the removal of biotin from the system, precluding further rounds of redistribution and recovery. This obstacle could be overcome by the use of lower-affinity biotin analogs, which would enable repeated cycles of coupling and uncoupling [66].

Other advantages of RAMP are that it uses an easily available, inexpensive, and nontoxic substance, biotin (i.e., a vitamin) and that it does not require any special instrumentation. Importantly, RAMP is not only useful for microscopic analyses but is also easily adaptable to biochemical applications. For example, cells expressing RAMP constructs in the absence or presence of biotin (and thus with different organelle distributions) can be analyzed in bulk by immunoblotting, pulse-chase analysis, subcellular fractionation, organelle proteomics, etc., without the need for rare chemicals or special equipment. Moreover, RAMP can be easily implemented for high-throughput screens. RAMP is thus a new, to our knowledge, addition to the toolbox for manipulating organelle positioning, which, because of its distinct features, ease of use, and versatility, may find broad application in the study of organelle biology.

## Methods

### Ethics statement

Animal procedures, including preparation of rat hippocampal neurons, were conducted under protocol #16–011 approved by the NICHD Animal Care and Use Committee, in adherence to the NIH Guide for the Care and Use of Laboratory Animals.

### Molecular cloning

DNA sequences encoding fragments from mouse KIF5A (KIF5A* = 1–559), mouse KIF5B (KIF5B* = 1–555), mouse KIF1A (KIF1A* = 1–405), human KIF13A (KIF13A* = 1–639), and human KIF17 (KIF17* = 1–509) were cloned into pmCherry-C1 (Clontech, Takara Bio, Mountain View, CA, USA). These fragments comprise the complete motor and neck domains and part of the coiled-coil domain required for dimerization and movement after recruitment to the target organelle [44]. DNA sequences encoding a 6×(glycine–serine) linker and streptavidin were cloned downstream of the truncated kinesins to complete the anterograde motor constructs (Fig 1B and S8B Fig). DNA sequences encoding streptavidin, followed by a 6×(glycine–serine) linker and a fragment from human KIFC1 (KIFC1* = 125–673), were cloned into pmCherry-N1 (Clontech) to create a retrograde motor construct (Fig 1B). We also engineered a BicD2-based dynein–dynactin adaptor by cloning DNA sequences encoding a mouse BicD2 fragment (BicD2* = 15–595) [29], followed by a linker (RTVSR) and streptavidin into pmCherry-C1 (Clontech). In addition, we made HA-epitope–tagged motor constructs (HA-KIF5B*-strep and strep-KIFC1*-HA) by restriction-free cloning to replace the mCherry protein with the HA epitope. The different cargo constructs carrying the SBP were generated by insertion of the SBP sequence (DEKTTGWRGGHVVEGLAGELEQLRARLEHHPQGQREP) between the organellar protein or protein fragment of interest and the fluorescent reporter protein (EGFP[A206K], a monomeric GFP variant [67], or mCherry) (Fig 1A). Full-length rat LAMP1 and fragments from human TOM20 (TOM20* = 1–30) [68], human PEX3 (PEX3* = 1–42) [29], and human VAP-A (VAP-A* = 118–249) cDNAs were used to target the SBP–GFP tandem to lysosomes, mitochondria, peroxisomes, and ER, respectively. The streptavidin and SBP peptide cDNAs were obtained from the bicistronic expression plasmid encoding the ER hook (streptavidin–KDEL) and the reporter ManII-SBP-GFP, respectively, described by Boncompain and colleagues [18]. Both plasmids were gifts from F. Perez (Institut Curie, Paris, France). All constructs were verified by DNA sequencing.

### Antibodies and other reagents

Rabbit anti-LAMTOR4 (cat. 13140) (1:200) was from Cell Signaling Technology (Danvers, MA, USA). Mouse anti-α-tubulin (DM1A; cat. T6199) (1:1,000) and D-biotin (cat. 47868) were from Sigma-Aldrich (St. Louis, MO, USA). Rabbit anti-TfR (cat. ab84036) (1:200) and rabbit anti-PMP70 (cat. ab3421) (1:500) were from Abcam (Cambridge, UK). Rabbit anti-Sec31A (cat. 17913-1-AP) (1:500) was from Proteintech (Rosemont, IL, USA). Mouse anti-calnexin (clone C8.B6; cat. MAB3126) (1:1,000) was from EMD Millipore (Burlington, MA, USA). Mouse anti-pan-neurofascin (external) (clone A12/18; cat. 75–172) (1:50) was from the University of California, Davis/NIH NeuroMab Facility (Davis, CA, USA). Mix-n-Stain-CF640R (cat. 92245) was from Biotium (Fremont, CA, USA) and was used to label the antibody against neurofascin. Mouse anti-LAMP1 (H4A3) (1:500) was from the Developmental Studies Hybridoma Bank (University of Iowa, Iowa City, IA, USA). Mouse anti-EEA1 (cat. 610457) (1:1,000) was from BD Biosciences (San Jose, CA, USA). All secondary antibodies (donkey anti-rabbit and anti-mouse conjugated with Alexa Fluor 488, 555, or 647) (1:1,000) were from Invitrogen (Carlsbad, CA, USA). NeutrAvidin Protein (cat. 31050), LysoTracker Blue DND-22 (cat. L7525), AF647-dextran (cat. D22914), MitoTracker Red CMXRos (cat. M7512), and DQ Red BSA (cat. D12051) were from Thermo Fisher Scientific (Waltham, MA, USA).

### Cell culture and transfection

HeLa, COS-7, and HT-1080 cells were cultured in Dulbecco’s Modified Eagle’s Medium (DMEM) (Corning, Corning, NY, USA) supplemented with 10% fetal bovine serum (Corning), 100 IU/mL penicillin and 100 μg/mL streptomycin (Corning), and 2 mM L-glutamine (Corning) in a 37°C incubator with 5% CO_2_ and 95% air. Primary cultures of rat hippocampal neurons were prepared at embryonic day 18 and cultured as previously described [69]. Cells were seeded in 24-well plates with glass coverslips or 8-well chambered cover glass (Cellvis, Mountain View, CA, USA). Plasmids were transfected using Lipofectamine 2000 (Thermo Fisher Scientific) following the manufacturer’s instructions. Unless otherwise stated, cells were analyzed 24 h after transfection.

### Fluorescence microscopy

For fixed-cell immunofluorescence microscopy experiments, coverslips were washed in PBS and fixed for 12 minutes in 4% paraformaldehyde and 4% sucrose in PBS at room temperature. Following fixation, coverslips were washed twice for 5 minutes in PBS, permeabilized for 15 minutes in 0.2% (v/v) Triton X-100, and blocked for 30 minutes in 0.2% porcine gelatin (Sigma-Aldrich) in PBS. Staining was carried out for 30 minutes at 37°C with different primary antibodies diluted in 0.2% gelatin in PBS (immunofluorescence buffer). Coverslips were then washed twice with PBS and incubated for 30 minutes at 37°C with Alexa-Fluor–conjugated secondary antibodies diluted in immunofluorescence buffer. Coverslips were next washed twice with PBS and mounted on slides using Fluoromount-G with or without DAPI (Electron Microscopy Sciences, Hatfield, PA, USA). Images were obtained on an inverted confocal laser scanning microscope (LSM880; Carl Zeiss, Oberkochen, Germany) fitted with a 63× 1.4 NA objective. Image analysis was performed using ImageJ (Rasband, W.S., ImageJ, U. S. National Institutes of Health, Bethesda, MD, USA, https://imagej.nih.gov/ij/, 1997–2016).

### Live-cell imaging

Live-cell imaging was conducted with an Eclipse Ti Microscope System (Nikon, Tokyo, Japan) equipped with an environmental chamber (temperature controlled at 37°C and CO_2_ at 5%) and NIS-Elements AR microscope imaging software. Cells were kept in microscopy medium (DMEM without phenol red, 1% bovine serum albumin [Sigma-Aldrich] and 25 mM HEPES [Corning] [pH 7.4], sterile filtered) during the entire imagining time. Spinning disk confocal images were taken with a Plan Apo VC 60× objective (NA 1.40) and a high-speed electron-multiplying charge-coupled device camera (Evolve 512; Photometrics, Tucson, AZ, USA). The focus on the sample was maintained using the Perfect Focus system. Dual-color imaging was done by fast switching of the excitation lasers, and images from green and red channels were aligned automatically. Laser power and exposure times were optimized depending on transfection efficiency and frame-rate acquisition.

### Electron microscopy

HeLa cells were grown in standard culture medium on borosilicate glass coverslips; fixed with 2.5% glutaraldehyde, 2% formaldehyde, and 2 mM CaCl_2_ in 0.1 M sodium cacodylate (pH 7.4) for 60 minutes; postfixed in 1% osmium tetroxide; and stained en bloc with aqueous 2% uranyl acetate overnight. Samples were dehydrated in increasing concentrations of ethanol, penetrated with epoxy resin, and subsequently polymerized at 65°C for 60 h. Coverslips were removed by hydrofluoric acid, and blocks were cut out and remounted on a microtome holder. Thin sections (80 nm) were cut parallel to the surface of the block, mounted on copper grids covered with formvar/carbon film, and stained with uranyl acetate and lead citrate. Samples were analyzed with an FEI Tecnai T20 microscope (Thermo Fisher Scientific) at 120 kV, and images were recorded with an AMT XR81 CCD camera (AMT, Woburn, MA, USA).

### Quantification of lysosome movement after RAMP reversal

Time-lapse microscopy analysis of lysosome movement after addition of biotin was performed as previously described [7], with some changes (S4 Fig). Control videos were generated by expression of only LAMP1-SBP-GFP, while RAMP experiments involved the coexpression of LAMP1-SBP-GFP with a streptavidin-tagged truncated kinesin plasmid (mCh-KIF5B*-strep or strep-KIFC1*-mCh). Unless otherwise indicated, addition of biotin corresponded to time 0. Frames from live-cell imaging were analyzed using ImageJ. Z-stacks for each frame were acquired and maximum intensity projections were generated before analysis. Individual cells were masked to exclude contributions from nearby cells. The Radial Profile Extended plug-in for ImageJ was used to calculate the radial profile (fluorescence intensity of the green channel as a function of radial distance—bin size was pixel size—where the center was set at the center of the nucleus) for each frame of the video. Next, distances were normalized, adjusting to “1” as the largest circle used in the radial profile generation (i.e., the most external circle that coincided with the border of the cell) and “0” as the center of the nucleus. Finally, from these profiles, we retrieved the radius required to include a fraction *f* of lysosomes by finding the minimal fractional distance at which the desired fraction of the total intensity was reached. We observed that in most of the videos analyzed, maximum difference between initial (time 0, no biotin) and final (normal distribution) average fractional distances was achieved when including 70%–95% of the total fluorescence (S4D Fig). This average fractional distance was plotted against time, and curves to analyze the recovery of the organelle distribution were used in Fig 3.

### Modeling of tug-of-war

The model, parameters, and simulations are described in detail in the Supporting Information (S1 Text).

### Analysis of lysosome movement in neurons

Movement of vesicles containing LAMP1-SBP-GFP protein was analyzed using ImageJ. Lines (1 pixel wide) were traced from the soma to axonal tips or from the soma to dendrite terminals, and the selection was straightened for all frames. Kymographs were generated by reslicing the straightened lines, followed by Z-projection. Anterograde or retrograde movement was manually determined from the kymographs by looking at the slopes of the generated lines, with negative and positive slopes representing anterograde and retrograde movement, respectively. Speed was calculated by manually measuring the initial and final position from lines in the kymographs to determine the path length in μm, divided by the elapsed time in seconds.

### Analysis of mitochondrial morphology

Mitochondrial morphology was analyzed as previously described [50]. In brief, confocal images for control (TOM20*-SBP-GFP only) and peripheral mitochondria (TOM20*-SBP-GFP and mCh-KIF5B*-strep coexpression) were acquired in HeLa cells transfected and fixed 24 h after transfection. The green channel (mitochondria) of these images was digitized using the ImageJ plug-in provided by Merrill and colleagues [50]. The following procedure was applied to each image: subtraction of background using a rolling radius of 50, followed by despeckle noise removal, contrast-limited histogram equalization (CLAHE) for enhancing local contrast (blocksize = 9 and maximum = 4) and a bandpass filtering (small = 0 and large = 256; suppress = none). Following autothresholding (method = Li), the “Analyze Particles” routine was used as originally provided with the macro to deliver the measurements. Six descriptors were obtained and analyzed: 1) length; 2) number of mitochondria; 3) area^2^ (average size of mitochondria weighted towards larger mitochondria); 4) aspect ratio (ratio of the major and short axis of an ellipse fitted to each mitochondria; the closer to 1 [minimum value], the rounder the mitochondria); 5) form factor (FF) (describes the mitochondria’s shape complexity; the minimum value of 1 indicates a perfect circle); and 6) area-weighted FF (variant of FF that averages with a bias towards larger mitochondria or mitochondrial networks). As suggested by the authors [50], mitochondrial morphology can be considered changed if at least three of the metrics show significant differences, and the trends are apparent to a blinded observer even when analyzing raw images.

### Material availability

The RAMP plasmids have been deposited in Addgene (IDs: 120163 to 120175). All other plasmids used in this study are available from the corresponding author upon request.

### Statistical analysis

Statistical analysis was performed using Prism 6 (GraphPad). Data are represented as mean ± SD (Fig 3C, and S7B Fig) or mean ± SEM (Fig 3D). Box-and-whisker plots show median, first and third quartiles, and maximum and minimum values (Figs 2F, 5D and 5F and S5D and S9D Figs). Summary data available as Supporting Information (S1 Data). *p*-values were determined using two-tailed Student *t* test for unpaired data or one-way ANOVA followed by Tukey’s test for multiple comparison of data in two or more groups (* *p* ≤ 0.05; ** *p* ≤ 0.01; *** *p* ≤ 0.001; **** *p* ≤ 0.0001. Nonsignificant differences were not highlighted for clarity in the figure).

## Supporting information

S1 Fig RAMP does not affect the microtubule cytoskeleton. Related to Fig 2. (A–F) Untransfected HeLa cells (A) or HeLa cells expressing the constructs indicated in the figure (B–F) were fixed, permeabilized, immunostained with antibody to α-tubulin, and imaged by confocal microscopy. Nuclei were stained with DAPI. Cell edges are outlined. Scale bar: 10 μm. Notice the integrity of the microtubule cytoskeleton in all cases. RAMP, reversible association with motor proteins.
(TIFF)

S2 Fig RAMP works in different cell lines and selectively affects the positioning of the target cargo. Related to Fig 2. (A,B) COS-7 (A) or HT-1080 (B) cells coexpressing the RAMP constructs indicated in the figure were fixed, permeabilized, and imaged by confocal microscopy. Nuclei were stained with DAPI. Cell edges are outlined. Notice the redistribution of lysosomes to the periphery or center of the cell. (C,D) HeLa cells coexpressing the RAMP constructs indicated in the figure were fixed, permeabilized, immunostained for endogenous TfR, and imaged by confocal microscopy. Nuclei were stained with DAPI. The rightmost image in the bottom row is a 3× magnification of the boxed area. Arrows indicate lysosomes. Notice the redistribution of lysosomes but not TfR endosomes in these cells. Scale bars: 10 μm. RAMP, reversible association with motor proteins; TfR, transferrin receptor.
(TIFF)

S3 Fig RAMP does not affect the function of lysosomes. Related to Fig 2. HeLa cells were co-transfected with plasmids encoding LAMP1-SBP-GFP and HA-KIF5B*-strep (A–C) or strep-KIFC1*-HA (D–F) and tested for various indicators of lysosomal function. Live cells were incubated for 30 minutes with 50 nM LysoTracker Blue DND-22 at 24 h after transfection (A,D), 16 h with 50 mg/mL AF647-dextran at 4 h after transfection (B,E), or 2 h with 10 μg/mL DQ-BSA at 24 h after transfection (C,F), all in complete medium at 37°C and 5% CO_2_. Cells were washed twice with PBS and fixed. Cell edges are outlined. Scale bar: 10 μm. Notice that clustering of lysosomes in the periphery or center of the cell does not affect lysosomal functions. AF647-dextran, Alexa Fluor 647-dextran; DQ-BSA, dye-quenched bovine serum albumin; GFP, green fluorescent protein; HA, hemagglutinin; KIF, kinesin superfamily; LAMP, lysosome-associated membrane protein; SBP, streptavidin-binding protein; strep, streptavidin.
(TIFF)

S4 Fig Analysis of lysosome redistribution in RAMP experiments. Related to Fig 3. (A) Schematic of the transfection and microscopy protocol for all the live-cell imagining experiments. HeLa cells were plated in 8-well chambered cover glass in complete medium. 18–24 h after seeding, cells were transfected with the plasmids of interest and allowed to express the constructs for 24 h. 15 minutes before acquisition, cells were washed twice with microscopy medium and kept in this medium before addition of biotin, all at 37°C. Once at the microscope, time-lapse microscopy videos were recorded (biotin addition was *t* = 0). (B) Z-stacks for each time frame were recorded. Maximum intensity Z-projections were generated and saved for each timeframe. (C) Using the Radial Profile Extended plug-in from ImageJ, Radial Distribution Profiles (fluorescence intensity as a function of radial distance, in which the center was set at the center of the nucleus) for each frame of the video were calculated. (D) These radial profiles were used to calculate the average fractional distance required to include a given fraction of lysosomes (*f*) (left graph), and select from those curves the one who presents the maximum difference (absolute value) between initial and final fractional distance to maximize the sensitivity in the changes observed (right graph). For more details, see Methods section. RAMP, reversible association with motor proteins.
(TIFF)

S5 Fig A BicD2-based RAMP construct behaves as a dominant-negative mutant. (A) Schematic representation of the dynein–dynactin adaptor protein BicD2 and the truncation previously used to move cargo to the center of the cell [9] repurposed for the RAMP method. Numbers correspond to the amino-acid sequence of the human protein. (B) HeLa cells coexpressing LAMP1-SBP-GFP and mCh-BicD2*-strep were analyzed by live-cell imaging after biotin addition (S3 Movie). Cell edges are outlined. Scale bar: 10 μm. Notice the accumulation of lysosomes at the cell center at time 0 and at peripheral cell protrusions at different times after the addition of biotin (arrows). The fact that lysosomes do not just return to their steady-state distribution but accumulate at cell protrusions indicates that mCh-BicD2*-strep has a dominant-negative effect on retrograde transport. (C) HeLa cells expressing mCh-BicD2*-strep plasmid alone for 24 h were fixed, permeabilized, and immunostained for endogenous LAMP1 and TfR. Cell edges are outlined. Nuclei were stained with DAPI. Arrows show cell protrusions. Scale bar: 10 μm. Notice that mCh-BicD2*-strep causes peripheral clustering of lysosomes and dispersal of endosomes by itself, confirming the dominant-negative effect on dynein–dynactin function. (D) Box-and-whisker plots represent the fractional distance distribution (*f* = 95%) of LAMP1- and TfR-positive vesicles in the conditions from panel C (see S4 Fig and Methods section for details). Summary data available as Supporting Information (S1_Data.xlsx). BicD2, bicaudal D homolog 2; CC, coiled coil; FP, fluorescent protein; GFP, green fluorescent protein; LAMP, lysosome-associated membrane protein; mCh, mCherry; RAMP, reversible association with motor proteins; SBP, streptavidin-binding protein; strep, streptavidin; TfR, transferrin receptor.
(TIFF)

S6 Fig Computational simulations of RAMP with lysosomes. Related to Fig 3. (A) Snapshots of the simulations of the release of lysosomes from the periphery of the cell at different times after release from the strep-tagged motor molecules KIF5B*. The big circle represents the border of the cell, while the inner smaller one represents the nucleus. Each point denotes a lysosome, representing the LAMP1-SBP-GFP–positive vesicles from experiments in Fig 3. (B) Snapshots of similar simulations performed as in (A) but in a condition in which lysosomes are released from the MTOC because of accumulation by strep-tagged motor construct KIFC1* and release with biotin. For more details on the computational model, check the S1 Text. GFP, green fluorescent protein; KIF, kinesin superfamily; LAMP, lysosome-associated membrane protein; MTOC, microtubule-organizing center; RAMP, reversible association with motor proteins; SBP, streptavidin-binding protein; strep, streptavidin.
(TIFF)

S7 Fig Application of RAMP to neuronal lysosomes. Related to Fig 4. (A) DIV5 rat hippocampal neurons were co-transfected with plasmids encoding LAMP1-SBP-GFP (left panel) and mCh-KIF5B*-strep (right panel) in the absence of biotin. The following day, neurons were fixed with 4% paraformaldehyde and imaged for GFP and mCherry. Arrowheads mark the trajectory of the axon. Arrows indicate the axon tip. Scale bar: 20 μm. The strips on the right show 50 μm of straightened axon terminal and 35-μm dendrite portion from the neuron on the left. (B) Quantification of speed (in μm/s) of 30 LAMP1-SBP-GFP–positive particles from at least 5 neurons per condition. Mean ± SD (in μm/s) from three independent experiments are (WT/RAMP) axon terminal (retrograde) (0.8 ± 0.1/0.63 ± 0.08); proximal axon (anterograde) (1.1 ± 0.2/1.1 ± 0.3); dendrites (anterograde) (0.8 ± 0.2/0.7 ± 0.2); and dendrites (retrograde) (0.9 ± 0.2/0.7 ± 0.3). Summary data available as Supporting Information (S1_Data.xlsx). DIV5, day in vitro 5; GFP, green fluorescent protein; KIF, kinesin superfamily; LAMP, lysosome-associated membrane protein; mCh, mCherry; RAMP, reversible association with motor proteins; SBP, streptavidin-binding protein; strep, streptavidin; WT, wild type.
(TIFF)

S8 Fig Properties of mitochondria subjected to RAMP with different motor constructs. Related to Fig 5. (A) HeLa cells were co-transfected with plasmids encoding TOM20*-SBP-GFP and HA-KIF5B*-strep or strep-KIFC1*-HA. At 24 h after transfection, cells were incubated for 30 minutes at 37°C, 5% CO_2_ with 25 nM MitoTracker Red CMXRos, washed twice in PBS, and immediately imaged at 0 and 10 minutes after addition of biotin. Notice that mitochondria stain with this mitochondrial marker regardless of their clustering to the periphery or center of the cell. (B) Schematic representation of alternative RAMP motor constructs based on the plus-end–directed kinesins KIF1A, KIF13A, and KIF17. Numbers correspond to the amino-acid sequences of the human proteins. (C) HeLa cells were co-transfected with plasmids encoding TOM20*-SBP-GFP and mCh-KIF1A*-strep, mCh-KIF13A*-strep, or mCh-KIF17*-strep. At 24 h after transfection, cells were imaged by confocal microscopy. All motor constructs caused accumulation of mitochondria at cell protrusions and a change of their morphology to rounder shape. Cell edges are outlined. Scale bars: 10 μm. CC, coiled coil; FHA, Forkhead-associated domain; FP, fluorescent protein; GFP, green fluorescent protein; HA, hemagglutinin; KIF, kinesin superfamily; mCh, mCherry; NC, neck coil domain; PH, Pleckstrin homology domain; RAMP, reversible association with motor proteins; SBP, streptavidin-binding protein; strep, streptavidin; TOM, translocase of the outer membrane.
(TIFF)

S9 Fig Manipulation of peroxisome distribution with RAMP. (A) Confocal microscopy of HeLa cells expressing PEX3*-SBP-GFP shows perfect colocalization with endogenous PMP70 and no colocalization with the early endosomal marker EEA1. (B) Coexpression of PEX3*-SBP-GFP and HA-KIF5B*-strep causes accumulation of peroxisomes at the cell periphery. (C) Coexpression of PEX3*-SBP-GFP and strep-KIFC1*-HA causes accumulation of peroxisomes in the perinuclear area of the cell. Nuclei were stained with DAPI. Rightmost panels in are 3.5× magnifications of the boxed areas. (D) Box-and-whisker plots represent the fractional distance distribution (*f* = 95%) of PEX3*-SBP-GFP–and PMP70-positive vesicles in the conditions from panels B and C (see S4 Fig and Methods section for details). Summary data available as Supporting Information (S1_Data.xlsx). (E,F) Reversal of peroxisome accumulation at the cell periphery (E) (see S8 Movie) and perinuclear area (F) (see S9 Movie) upon addition of biotin. Cell edges are outlined. Scale bars: 10 μm. EEA1, early endosome antigen 1; GFP, green fluorescent protein; HA, hemagglutinin; KIF, kinesin superfamily; PEX, peroxin; PMP70, peroxisomal membrane protein 70; RAMP, reversible association with motor proteins; SBP, streptavidin-binding protein; strep, streptavidin.
(TIFF)

S1 Movie RAMP of lysosomes from the periphery of the cell. Related to Fig 3. LAMP1-SBP-GFP (green) and mCh-KIF5B*-strep (red) were coexpressed in HeLa cells in the absence of biotin. The video (10 fps) shows the release of lysosomes from the periphery by the addition of biotin to the medium (time = 0). The movie was sequentially recorded every 1 minute for 45 minutes on a spinning disk confocal microscope. Each frame corresponds to the maximum intensity projection of a Z-stack for each channel. Right panel shows the merged green and red channels. GFP, green fluorescent protein; KIF, kinesin superfamily; LAMP, lysosome-associated membrane protein; mCh, mCherry; RAMP, reversible association with motor proteins; SBP, streptavidin-binding protein; strep, streptavidin.
(MOV)

S2 Movie RAMP of lysosomes from the center of the cell. Related to Fig 3. Strep-KIFC1*-mCh (red) and LAMP1-SBP-GFP (green) were coexpressed in HeLa cells in the absence of biotin. The video (10 fps) shows the release of lysosomes from the perinuclear area by the addition of biotin to the medium (time = 0). The movie was sequentially recorded every 1 minute for 45 minutes on a spinning disk confocal microscope. Each frame corresponds to the maximum intensity projection of a Z-stack for each channel. Right panel shows the merged green and red channels. GFP, green fluorescent protein; KIF, kinesin superfamily; LAMP, lysosome-associated membrane protein; mCh, mCherry; RAMP, reversible association with motor proteins; SBP, streptavidin-binding protein; strep, streptavidin.
(MOV)

S3 Movie BicD2-based RAMP of lysosomes from the center of the cell. Related to S5 Fig. LAMP1-SBP-GFP (green) and mCh-BICD2*-strep (red) were coexpressed in HeLa cells in the absence of biotin. The video (10 fps) shows the release of lysosomes from the perinuclear area and subsequent accumulation in the tips of the cell by the addition of biotin to the medium (time = 0). The movie was sequentially recorded every 1 minute for 70 minutes on a spinning disk confocal microscope. Each frame corresponds to the maximum intensity projection of a Z-stack for each channel. Right panel shows the merged green and red channels. BicD2, bicaudal D homolog 2; GFP, green fluorescent protein; LAMP, lysosome-associated membrane protein; mCh, mCherry; RAMP, reversible association with motor proteins; SBP, streptavidin-binding protein; strep, streptavidin.
(MOV)

S4 Movie RAMP of mitochondria from the periphery of the cell. Related to Fig 5. TOM20*-SBP-GFP (green) and mCh-KIF5B*-strep (red) were coexpressed in HeLa cells in the absence of biotin. The video (10 fps) shows the change in morphology in mitochondria at *t* = 0 and their release from the periphery by the addition of biotin to the medium (time = 0). The movie was sequentially recorded every 1 minute for 45 minutes on a spinning disk confocal microscope. Each frame corresponds to the maximum intensity projection of a Z-stack for each channel. Right panel shows the merged green and red channels. GFP, green fluorescent protein; KIF, kinesin superfamily; mCh, mCherry; RAMP, reversible association with motor proteins; SBP, streptavidin-binding protein; strep, streptavidin; TOM, translocase of the outer membrane.
(MOV)

S5 Movie RAMP of mitochondria from the center of the cell. Related to Fig 5. TOM20*-SBP-GFP (green) and strep-KIFC1*-mCh (red) were coexpressed in HeLa cells in the absence of biotin. The video (10 fps) shows the accumulation of mitochondria at *t* = 0 and their release from the center of the cell by the addition of biotin to the medium (time = 0). The movie was sequentially recorded every 1 minute for 45 minutes on a spinning disk confocal microscope. Each frame corresponds to the maximum intensity projection of a Z-stack for each channel. Right panel shows the merged green and red channels. GFP, green fluorescent protein; KIF, kinesin superfamily; mCh, mCherry; RAMP, reversible association with motor proteins; SBP, streptavidin-binding protein; strep, streptavidin; TOM, translocase of the outer membrane.
(MOV)

S6 Movie RAMP of an ER-resident protein from the periphery of the cell. Related to Fig 6. GFP-SBP-VAP-A* (green) and mCh-KIF5B*-strep (red) were coexpressed in HeLa cells in the absence of biotin. The video (10 fps) shows the redistribution of the VAP-A* construct at *t* = 0 and its release by the addition of biotin to the medium (time = 0). The movie was sequentially recorded every 1 minute for 45 minutes on a spinning disk confocal microscope. Each frame corresponds to the maximum intensity projection of a Z-stack for each channel. Right panel shows the merged green and red channels. ER, endoplasmic reticulum; GFP, green fluorescent protein; KIF, kinesin superfamily; mCh, mCherry; RAMP, reversible association with motor proteins; SBP, streptavidin-binding protein; strep, streptavidin; VAP, VAMP-associated protein.
(MOV)

S7 Movie RAMP of an ER-resident protein from the perinuclear area of the cell. Related to Fig 6. GFP-SBP-VAP-A* (green) and strep-KIFC1*-mCh (red) were coexpressed in HeLa cells in the absence of biotin. The video (10 fps) shows the redistribution of the VAP-A* construct at *t* = 0 from the center to the rest of the ER network by the addition of biotin to the medium (time = 0). The movie was sequentially recorded every 1 minute for 45 minutes on a spinning disk confocal microscope. Each frame corresponds to the maximum intensity projection of a Z-stack for each channel. Right panel shows the merged green and red channels. ER, endoplasmic reticulum; GFP, green fluorescent protein; KIF, kinesin superfamily; mCh, mCherry; RAMP, reversible association with motor proteins; SBP, streptavidin-binding protein; strep, streptavidin; VAP, VAMP-associated proteinXXX.
(MOV)

S8 Movie RAMP of peroxisomes from the periphery of the cell. Related to S9 Fig. PEX3*-SBP-GFP (green) and mCh-KIF5B*-strep (red) were coexpressed in HeLa cells in the absence of biotin. The video (10 fps) shows the release of peroxisomes from the periphery by the addition of biotin to the medium (time = 0). The movie was sequentially recorded every 1 minute for 43 minutes on a spinning disk confocal microscope. Each frame corresponds to the maximum intensity projection of a Z-stack for each channel. Right panel shows the merged green and red channels. GFP, green fluorescent protein; KIF, kinesin superfamily; mCh, mCherry; PEX, peroxin; RAMP, reversible association with motor proteins; SBP, streptavidin-binding protein; strep, streptavidin.
(MOV)

S9 Movie RAMP of peroxisomes from the center of the cell. Related to S9 Fig. PEX3*-SBP-GFP (green) and strep-KIFC1*-mCh (red) were coexpressed in HeLa cells in the absence of biotin. The video (10 fps) shows the release of peroxisomes from the center of the cell by the addition of biotin to the medium (time = 0). The movie was sequentially recorded every 1 minute for 45 minutes on a spinning disk confocal microscope. Each frame corresponds to the maximum intensity projection of a Z-stack for each channel. Right panel shows the merged green and red channels. GFP, green fluorescent protein; KIF, kinesin superfamily; mCh, mCherry; PEX, peroxin; RAMP, reversible association with motor proteins; SBP, streptavidin-binding protein; strep, streptavidin.
(MOV)

S1 Text Tug-of-war simulations on release of lysosomes. Related to Fig 3 and S6 Fig. Model description and parameters used in the simulations of the tug-of-war during RAMP release of lysosomes. RAMP, reversible association with motor proteins.
(PDF)

S1 Data Raw data and summary statistics underlying Figs 2F, 3C, 3D, 5D, 5F, S4D, S5D, S7B and S9D. (XLSX)

## Abbreviations

AF647: dextran, Alexa Fluor 647-dextran
AIS: axon initial segment
BicD2: bicaudal D homolog 2
CIB1: cryptochrome-interacting basic helix–loop–helix
CLAHE: contrast-limited histogram equalization
COP1: constitutively photomorphogenic 1
COS-7: CV-1 in Origin with SV40 genes-7
CRY2: cryptochrome 2
CUTE: controlled unmasking of targeting elements
DIV5: day in vitro 5
DLG1: discs large homolog 1
DMEM: Dulbecco’s Modified Eagle’s Medium
DQ-BSA: dye-quenched bovine serum albumin
EEA1: early endosome antigen 1
ePDZ: engineered PDZ domain
ER: endoplasmic reticulum
ERES: ER exit site
FF: form factor
FKBP: FK506 binding protein
FRB: FKBP-rapamycin binding domain
GAI: gibberellin insensitive
GFP: green fluorescent protein
GID1: gibberellin insensitive dwarf1
HA: hemagglutinin
JIP: JNK-interacting protein
KIF: kinesin superfamily
LAMP: lysosome-associated membrane protein
LAMTOR4: late endosomal/lysosomal adaptor and MAPK and MTOR activator 4
LOVpep: light–oxygen–voltage–peptide domain
MAP: microtubule-associated protein
mCh: mCherry
MTOC: microtubule-organizing center
PEX: peroxin
PMP70: peroxisomal membrane protein 70
PSD95: postsynaptic density protein
PDZ: PSD95, DLG1, and ZO-1
RAMP: reversible association with motor proteins
RILP: Rab-interacting lysosomal protein
RUSH: retention using selective hooks
SBP: streptavidin-binding peptide
Sec31A: secretory protein 31A
strep: streptavidin
TfR: transferrin receptor
TOM: translocase of the outer membrane
UVR8: UV resistance locus 8
VAP: VAMP-associated protein
VAMP: vesicle-associated membrane protein
WT: wild type
ZO-1: zonula occludens 1 protein
