# Supplementary figures and images for: Reversible association with motor proteins (RAMP): A streptavidin-based method to manipulate organelle positioning
Source: PLoS Biol. 2019 May 17;17(5):e3000279. doi: 10.1371/journal.pbio.3000279 (PMC6542540; doi:10.1371/journal.pbio.3000279)

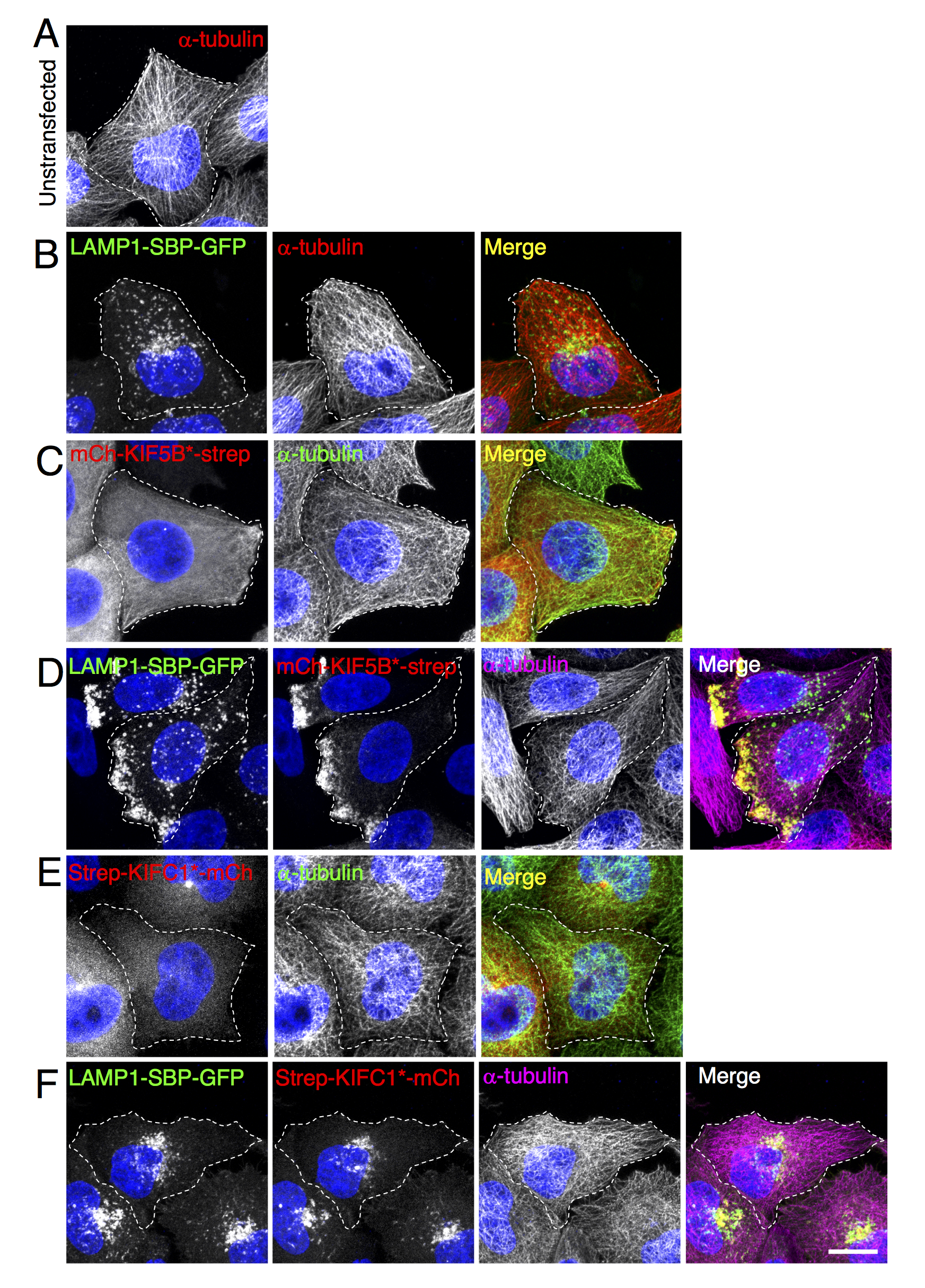

Supplement: S1 Fig — Related to Fig 2. (A–F) Untransfected HeLa cells (A) or HeLa cells expressing the constructs indicated in the figure (B–F) were fixed, permeabilized, immunostained with antibody to α-tubulin, and imaged by confocal microscopy. Nuclei were stained with DAPI. Cell edges are outlined. Scale bar: 10 μm. Notice the integrity of the microtubule cytoskeleton in all cases. RAMP, reversible association with motor proteins. (TIFF) [file pbio.3000279.s001.tiff]

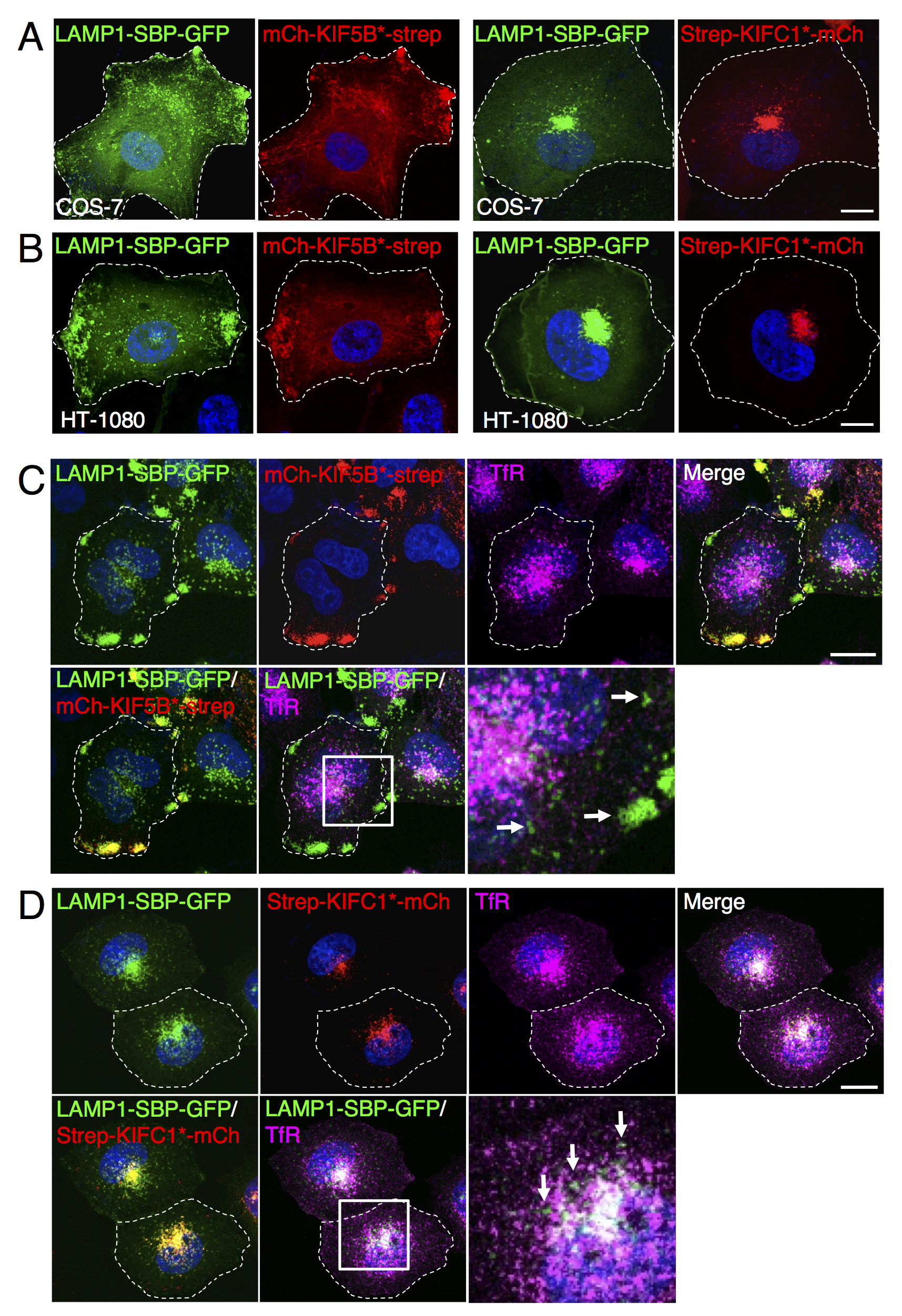

Supplement: S2 Fig — Related to Fig 2. (A,B) COS-7 (A) or HT-1080 (B) cells coexpressing the RAMP constructs indicated in the figure were fixed, permeabilized, and imaged by confocal microscopy. Nuclei were stained with DAPI. Cell edges are outlined. Notice the redistribution of lysosomes to the periphery or center of the cell. (C,D) HeLa cells coexpressing the RAMP constructs indicated in the figure were fixed, permeabilized, immunostained for endogenous TfR, and imaged by confocal microscopy. Nuclei were stained with DAPI. The rightmost image in the bottom row is a 3× magnification of the boxed area. Arrows indicate lysosomes. Notice the redistribution of lysosomes but not TfR endosomes in these cells. Scale bars: 10 μm. RAMP, reversible association with motor proteins; TfR, transferrin receptor. (TIFF) [file pbio.3000279.s002.tiff]

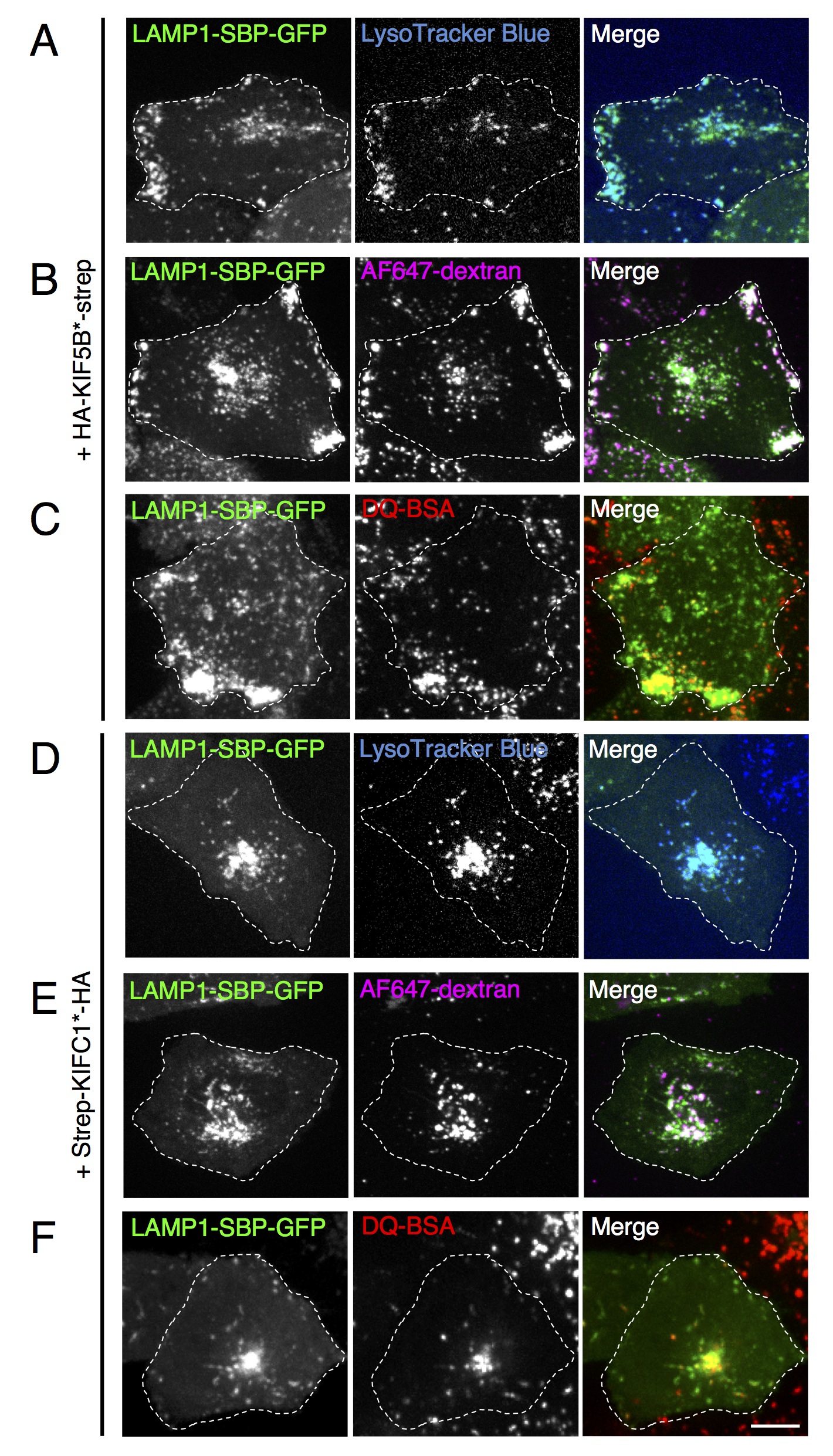

Supplement: S3 Fig — Related to Fig 2. HeLa cells were co-transfected with plasmids encoding LAMP1-SBP-GFP and HA-KIF5B*-strep (A–C) or strep-KIFC1*-HA (D–F) and tested for various indicators of lysosomal function. Live cells were incubated for 30 minutes with 50 nM LysoTracker Blue DND-22 at 24 h after transfection (A,D), 16 h with 50 mg/mL AF647-dextran at 4 h after transfection (B,E), or 2 h with 10 μg/mL DQ-BSA at 24 h after transfection (C,F), all in complete medium at 37°C and 5% CO2. Cells were washed twice with PBS and fixed. Cell edges are outlined. Scale bar: 10 μm. Notice that clustering of lysosomes in the periphery or center of the cell does not affect lysosomal functions. AF647-dextran, Alexa Fluor 647-dextran; DQ-BSA, dye-quenched bovine serum albumin; GFP, green fluorescent protein; HA, hemagglutinin; KIF, kinesin superfamily; LAMP, lysosome-associated membrane protein; SBP, streptavidin-binding protein; strep, streptavidin. (TIFF) [file pbio.3000279.s003.tiff]

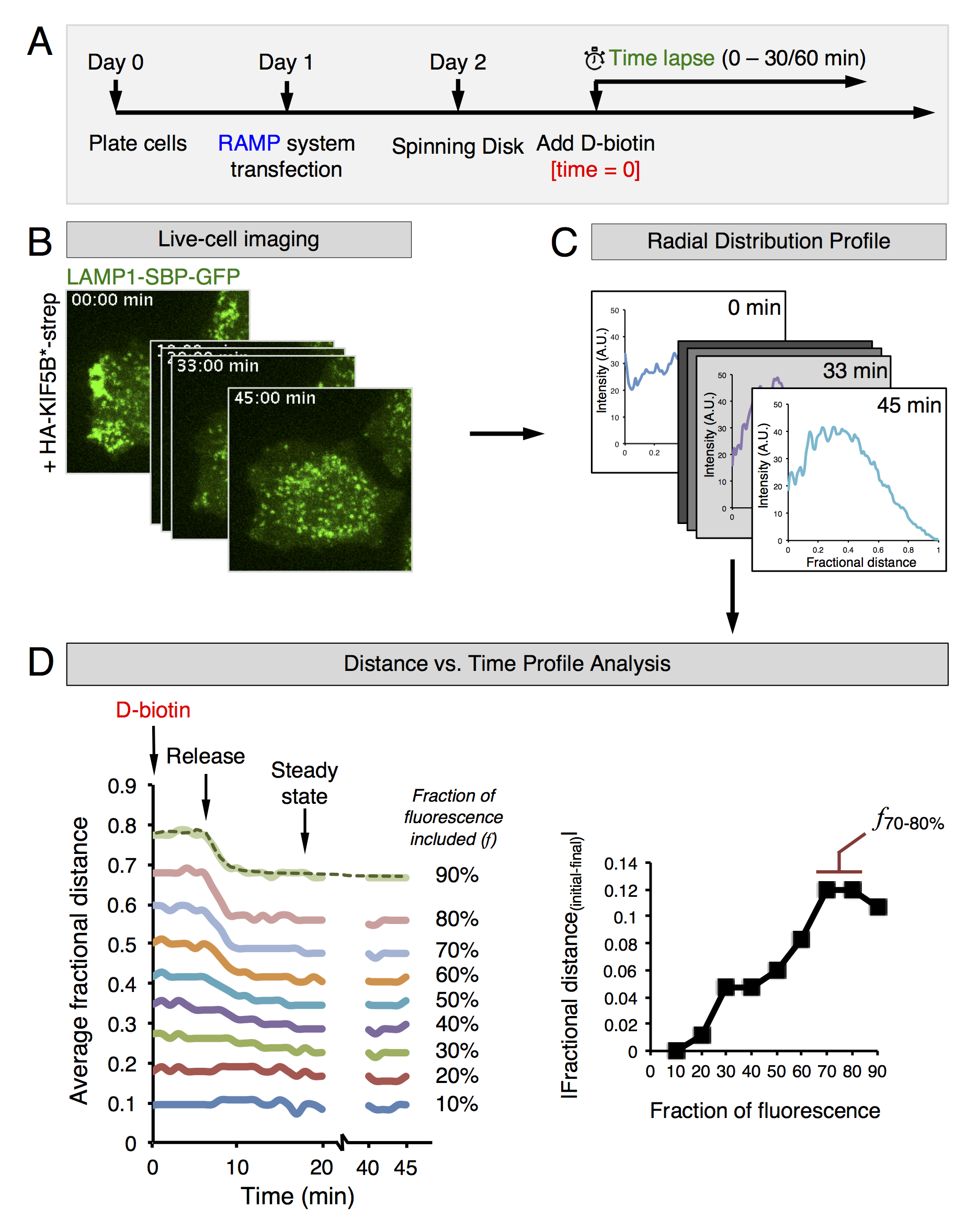

Supplement: S4 Fig — Related to Fig 3. (A) Schematic of the transfection and microscopy protocol for all the live-cell imagining experiments. HeLa cells were plated in 8-well chambered cover glass in complete medium. 18–24 h after seeding, cells were transfected with the plasmids of interest and allowed to express the constructs for 24 h. 15 minutes before acquisition, cells were washed twice with microscopy medium and kept in this medium before addition of biotin, all at 37°C. Once at the microscope, time-lapse microscopy videos were recorded (biotin addition was t = 0). (B) Z-stacks for each time frame were recorded. Maximum intensity Z-projections were generated and saved for each timeframe. (C) Using the Radial Profile Extended plug-in from ImageJ, Radial Distribution Profiles (fluorescence intensity as a function of radial distance, in which the center was set at the center of the nucleus) for each frame of the video were calculated. (D) These radial profiles were used to calculate the average fractional distance required to include a given fraction of lysosomes (f) (left graph), and select from those curves the one who presents the maximum difference (absolute value) between initial and final fractional distance to maximize the sensitivity in the changes observed (right graph). For more details, see Methods section. RAMP, reversible association with motor proteins. (TIFF) [file pbio.3000279.s004.tiff]

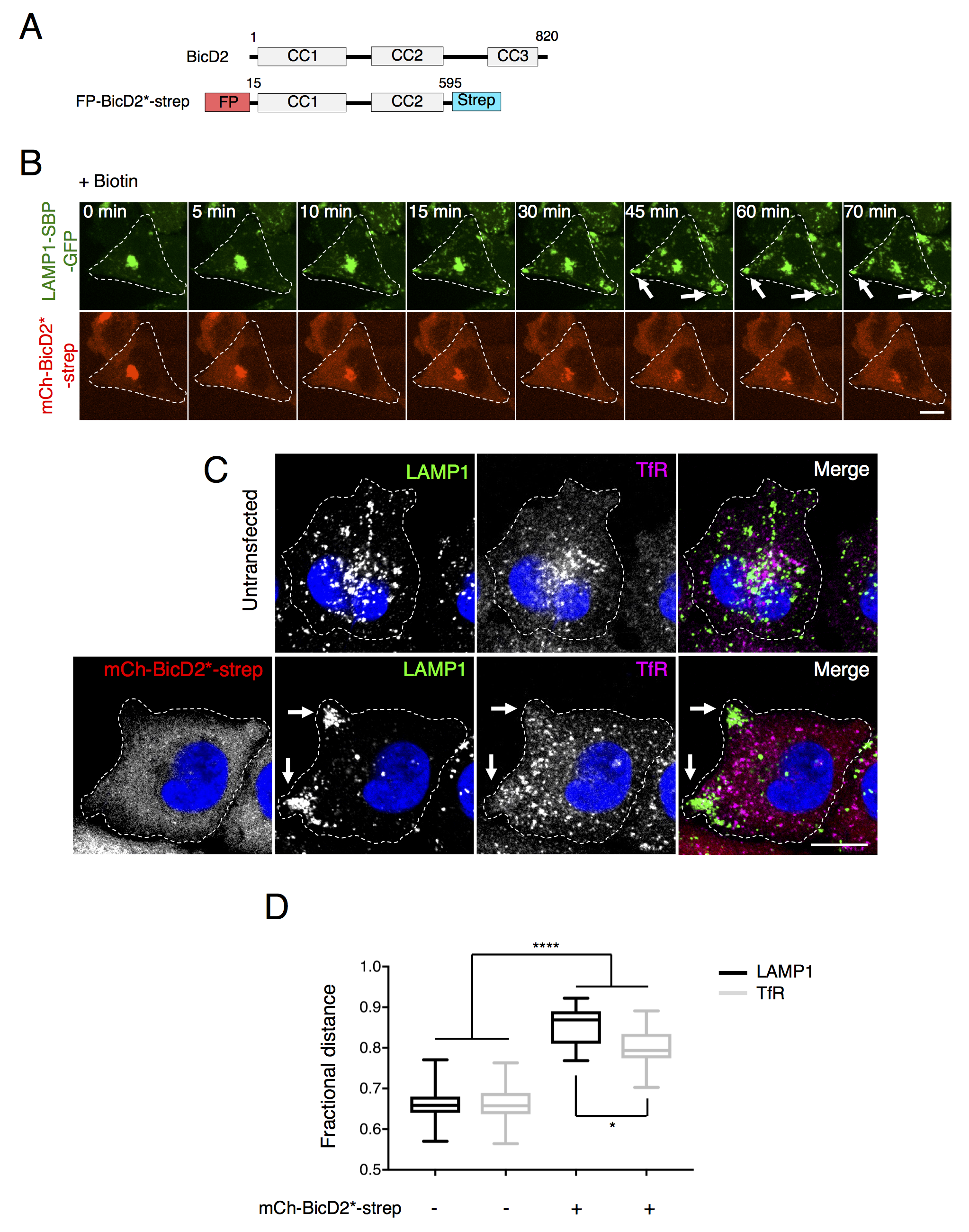

Supplement: S5 Fig — (A) Schematic representation of the dynein–dynactin adaptor protein BicD2 and the truncation previously used to move cargo to the center of the cell [9] repurposed for the RAMP method. Numbers correspond to the amino-acid sequence of the human protein. (B) HeLa cells coexpressing LAMP1-SBP-GFP and mCh-BicD2*-strep were analyzed by live-cell imaging after biotin addition (S3 Movie). Cell edges are outlined. Scale bar: 10 μm. Notice the accumulation of lysosomes at the cell center at time 0 and at peripheral cell protrusions at different times after the addition of biotin (arrows). The fact that lysosomes do not just return to their steady-state distribution but accumulate at cell protrusions indicates that mCh-BicD2*-strep has a dominant-negative effect on retrograde transport. (C) HeLa cells expressing mCh-BicD2*-strep plasmid alone for 24 h were fixed, permeabilized, and immunostained for endogenous LAMP1 and TfR. Cell edges are outlined. Nuclei were stained with DAPI. Arrows show cell protrusions. Scale bar: 10 μm. Notice that mCh-BicD2*-strep causes peripheral clustering of lysosomes and dispersal of endosomes by itself, confirming the dominant-negative effect on dynein–dynactin function. (D) Box-and-whisker plots represent the fractional distance distribution (f = 95%) of LAMP1- and TfR-positive vesicles in the conditions from panel C (see S4 Fig and Methods section for details). Summary data available as Supporting Information (S1_Data.xlsx). BicD2, bicaudal D homolog 2; CC, coiled coil; FP, fluorescent protein; GFP, green fluorescent protein; LAMP, lysosome-associated membrane protein; mCh, mCherry; RAMP, reversible association with motor proteins; SBP, streptavidin-binding protein; strep, streptavidin; TfR, transferrin receptor. (TIFF) [file pbio.3000279.s005.tiff]

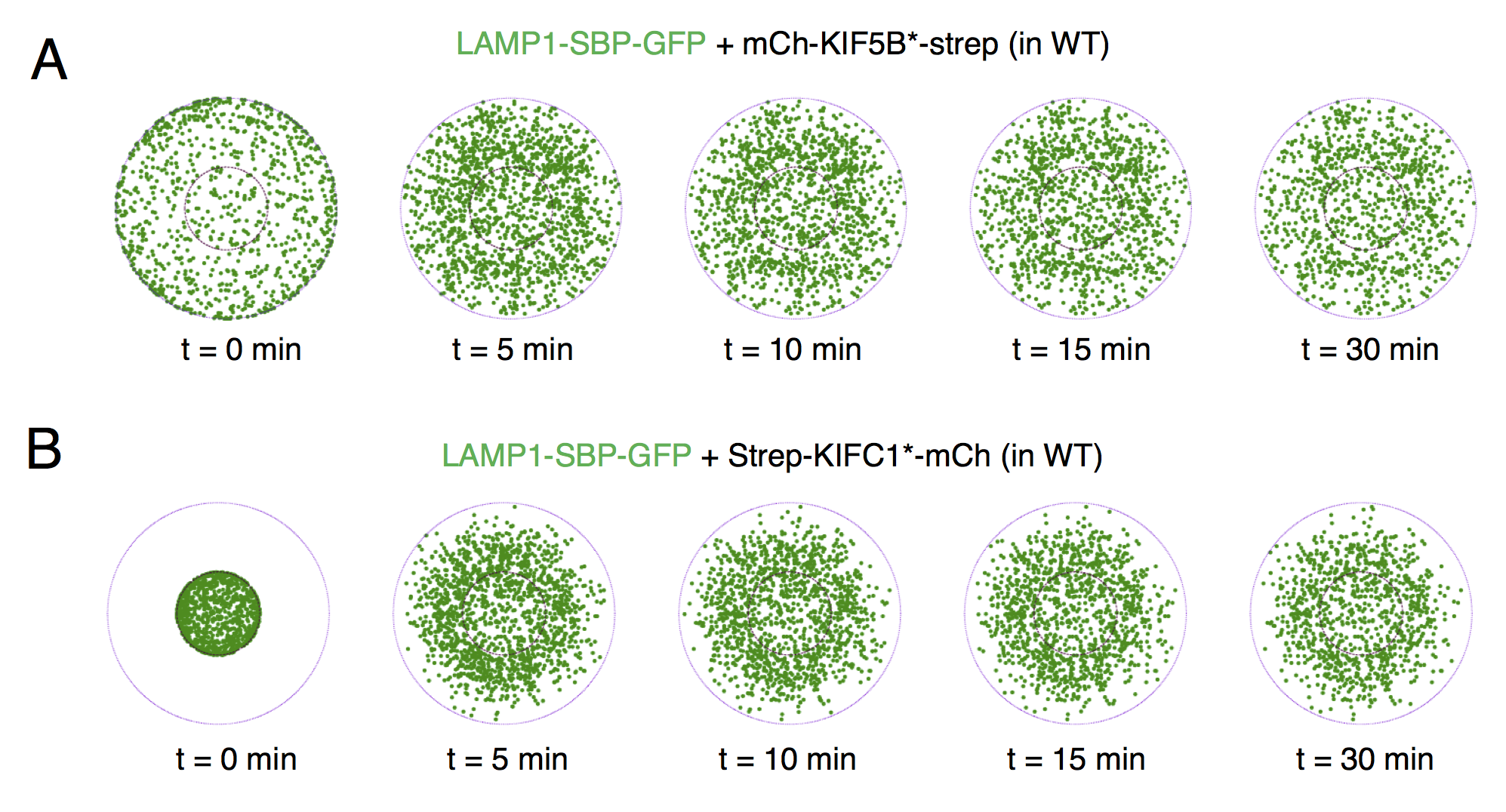

Supplement: S6 Fig — Related to Fig 3. (A) Snapshots of the simulations of the release of lysosomes from the periphery of the cell at different times after release from the strep-tagged motor molecules KIF5B*. The big circle represents the border of the cell, while the inner smaller one represents the nucleus. Each point denotes a lysosome, representing the LAMP1-SBP-GFP–positive vesicles from experiments in Fig 3. (B) Snapshots of similar simulations performed as in (A) but in a condition in which lysosomes are released from the MTOC because of accumulation by strep-tagged motor construct KIFC1* and release with biotin. For more details on the computational model, check the S1 Text. GFP, green fluorescent protein; KIF, kinesin superfamily; LAMP, lysosome-associated membrane protein; MTOC, microtubule-organizing center; RAMP, reversible association with motor proteins; SBP, streptavidin-binding protein; strep, streptavidin. (TIFF) [file pbio.3000279.s006.tiff]

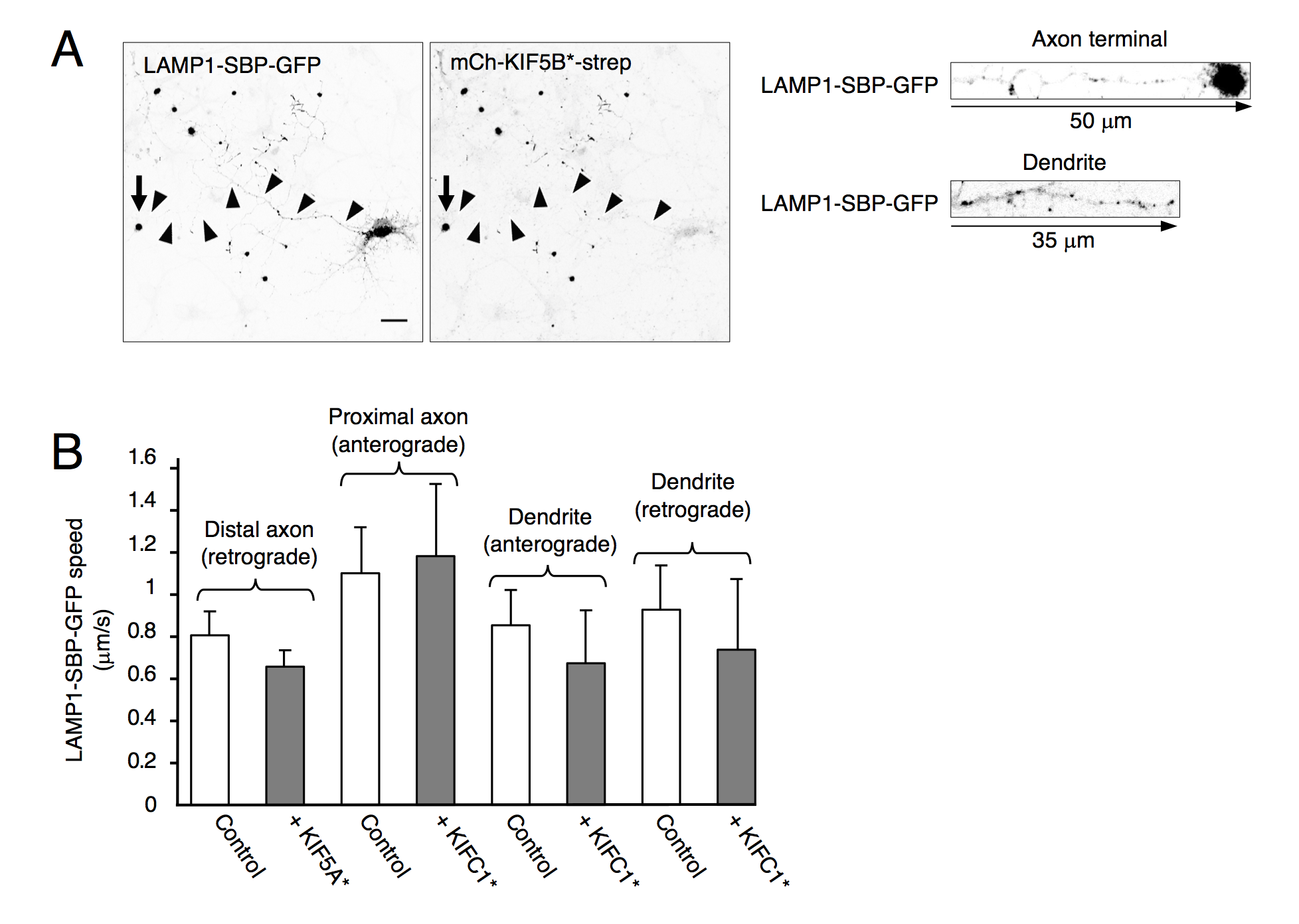

Supplement: S7 Fig — Related to Fig 4. (A) DIV5 rat hippocampal neurons were co-transfected with plasmids encoding LAMP1-SBP-GFP (left panel) and mCh-KIF5B*-strep (right panel) in the absence of biotin. The following day, neurons were fixed with 4% paraformaldehyde and imaged for GFP and mCherry. Arrowheads mark the trajectory of the axon. Arrows indicate the axon tip. Scale bar: 20 μm. The strips on the right show 50 μm of straightened axon terminal and 35-μm dendrite portion from the neuron on the left. (B) Quantification of speed (in μm/s) of 30 LAMP1-SBP-GFP–positive particles from at least 5 neurons per condition. Mean ± SD (in μm/s) from three independent experiments are (WT/RAMP) axon terminal (retrograde) (0.8 ± 0.1/0.63 ± 0.08); proximal axon (anterograde) (1.1 ± 0.2/1.1 ± 0.3); dendrites (anterograde) (0.8 ± 0.2/0.7 ± 0.2); and dendrites (retrograde) (0.9 ± 0.2/0.7 ± 0.3). Summary data available as Supporting Information (S1_Data.xlsx). DIV5, day in vitro 5; GFP, green fluorescent protein; KIF, kinesin superfamily; LAMP, lysosome-associated membrane protein; mCh, mCherry; RAMP, reversible association with motor proteins; SBP, streptavidin-binding protein; strep, streptavidin; WT, wild type. (TIFF) [file pbio.3000279.s007.tiff]

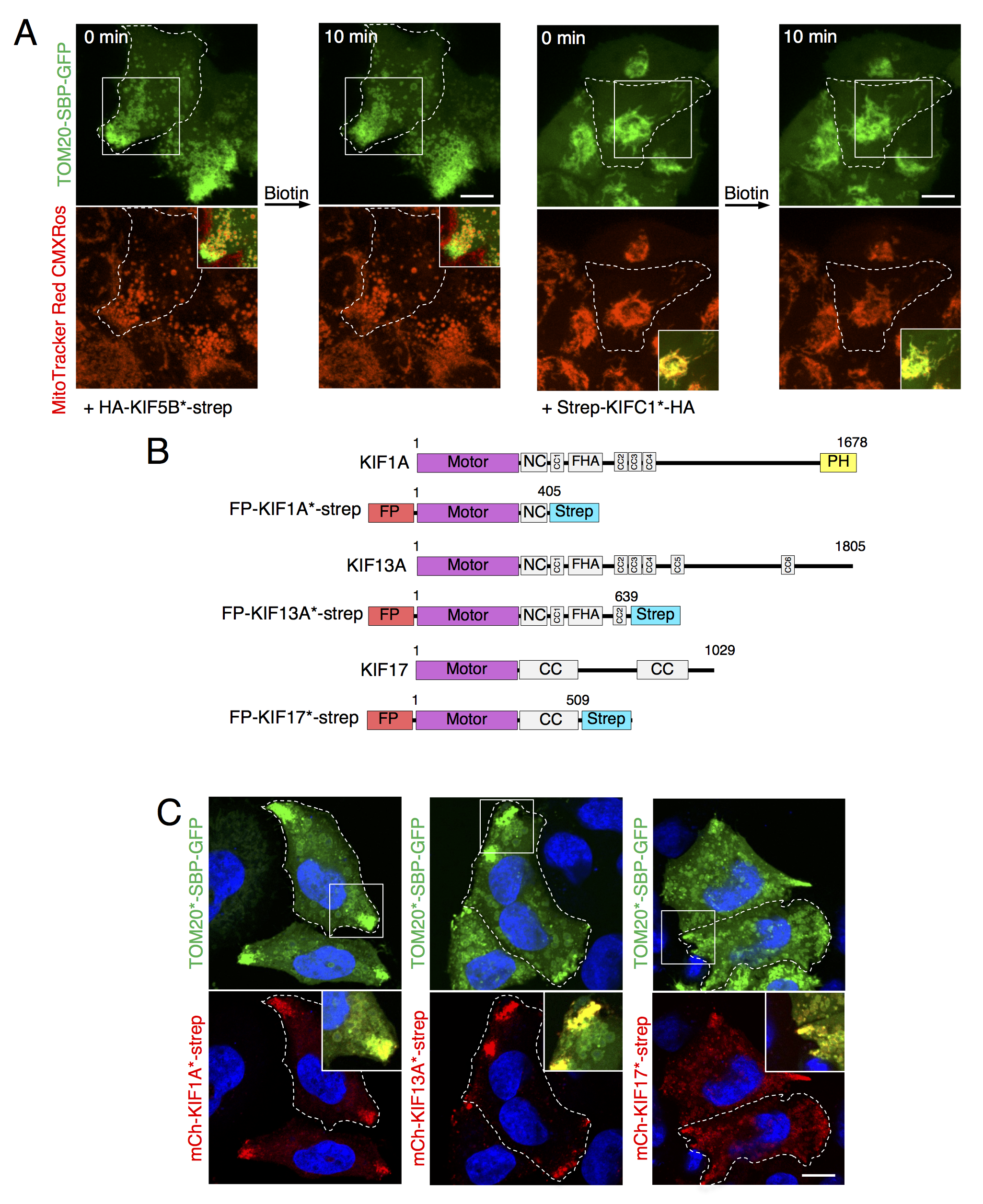

Supplement: S8 Fig — Related to Fig 5. (A) HeLa cells were co-transfected with plasmids encoding TOM20*-SBP-GFP and HA-KIF5B*-strep or strep-KIFC1*-HA. At 24 h after transfection, cells were incubated for 30 minutes at 37°C, 5% CO2 with 25 nM MitoTracker Red CMXRos, washed twice in PBS, and immediately imaged at 0 and 10 minutes after addition of biotin. Notice that mitochondria stain with this mitochondrial marker regardless of their clustering to the periphery or center of the cell. (B) Schematic representation of alternative RAMP motor constructs based on the plus-end–directed kinesins KIF1A, KIF13A, and KIF17. Numbers correspond to the amino-acid sequences of the human proteins. (C) HeLa cells were co-transfected with plasmids encoding TOM20*-SBP-GFP and mCh-KIF1A*-strep, mCh-KIF13A*-strep, or mCh-KIF17*-strep. At 24 h after transfection, cells were imaged by confocal microscopy. All motor constructs caused accumulation of mitochondria at cell protrusions and a change of their morphology to rounder shape. Cell edges are outlined. Scale bars: 10 μm. CC, coiled coil; FHA, Forkhead-associated domain; FP, fluorescent protein; GFP, green fluorescent protein; HA, hemagglutinin; KIF, kinesin superfamily; mCh, mCherry; NC, neck coil domain; PH, Pleckstrin homology domain; RAMP, reversible association with motor proteins; SBP, streptavidin-binding protein; strep, streptavidin; TOM, translocase of the outer membrane. (TIFF) [file pbio.3000279.s008.tiff]

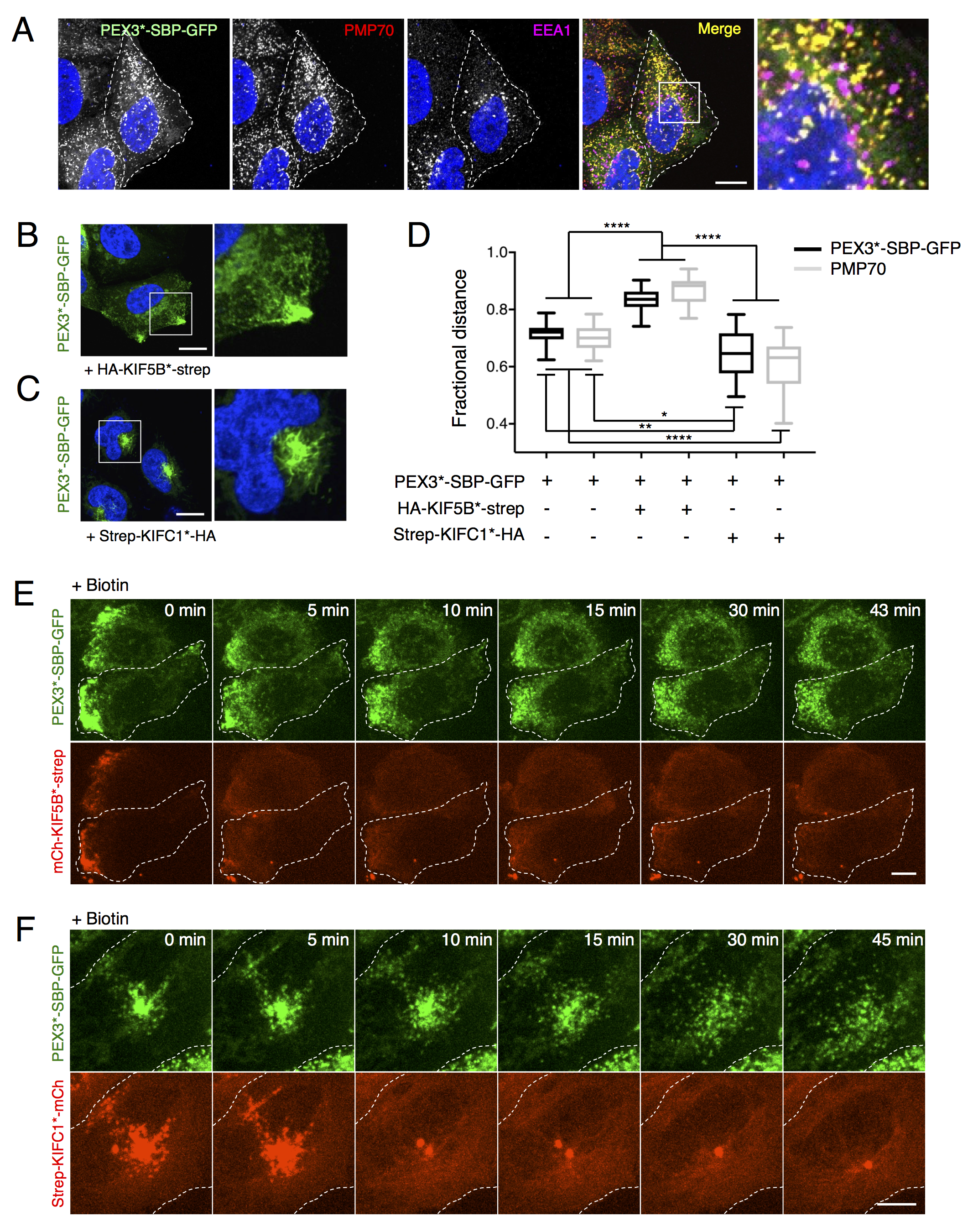

Supplement: S9 Fig — (A) Confocal microscopy of HeLa cells expressing PEX3*-SBP-GFP shows perfect colocalization with endogenous PMP70 and no colocalization with the early endosomal marker EEA1. (B) Coexpression of PEX3*-SBP-GFP and HA-KIF5B*-strep causes accumulation of peroxisomes at the cell periphery. (C) Coexpression of PEX3*-SBP-GFP and strep-KIFC1*-HA causes accumulation of peroxisomes in the perinuclear area of the cell. Nuclei were stained with DAPI. Rightmost panels in are 3.5× magnifications of the boxed areas. (D) Box-and-whisker plots represent the fractional distance distribution (f = 95%) of PEX3*-SBP-GFP–and PMP70-positive vesicles in the conditions from panels B and C (see S4 Fig and Methods section for details). Summary data available as Supporting Information (S1_Data.xlsx). (E,F) Reversal of peroxisome accumulation at the cell periphery (E) (see S8 Movie) and perinuclear area (F) (see S9 Movie) upon addition of biotin. Cell edges are outlined. Scale bars: 10 μm. EEA1, early endosome antigen 1; GFP, green fluorescent protein; HA, hemagglutinin; KIF, kinesin superfamily; PEX, peroxin; PMP70, peroxisomal membrane protein 70; RAMP, reversible association with motor proteins; SBP, streptavidin-binding protein; strep, streptavidin. (TIFF) [file pbio.3000279.s009.tiff]
