# Supplementary material for: Reversible association with motor proteins (RAMP): A streptavidin-based method to manipulate organelle positioning
Source: PLoS Biol. 2019 May 17;17(5):e3000279. doi: 10.1371/journal.pbio.3000279 (PMC6542540; doi:10.1371/journal.pbio.3000279)
Supplement: S1 Text — Related to Fig 3 and S6 Fig. Model description and parameters used in the simulations of the tug-of-war during RAMP release of lysosomes. RAMP, reversible association with motor proteins. (PDF) [file pbio.3000279.s019.pdf]

Models of Organelle Positioning

To understand the organelle positioning by opposite polarity motors which undergo tug-of-war during cargo transport, we developed a computational model based on a bidirectional cargo transport model first proposed by Muller *et al.* (2008) [1]. Their model assumes that the presence of opposite polarity motors induces a load force, and that load force is shared equally among the bound motors belonging to the same species.

In the bidirectional cargo transport model by Muller *et al*. [1], a cargo is transported by a team of N_+ microtubule plus-end-directed and N_- microtubule minus-directed motors which attach and detach from a microtubule stochastically with given on and off rates. The force-velocity relation for the motors was assumed to be a linear function of the applied load, and force-dissociation rate of the motors was assumed to be an exponentially increasing function of the load. When bound to the microtubule, the motor walks forward with the velocity v_F, which decreases linearly with the external force and reaches zero at the stall force F_s. Under superstall external force ($F > F_s$), the motor walks backward slowly with backward velocity v_B

$$v(F) = \begin{cases} v_F(1 - \frac{F}{F_S})\ ,\ F < F_s \\ v_B(1 - \frac{F}{F_S}),\ F \geq F_s \end{cases} \tag{1}$$

The rates for unbinding of one of the bound motors from microtubule and for binding of an additional unbound motor to microtubule are found based on the assumption that (i) the presence of opposing motors induces a load force, and (ii) each plus-end directed motor feels the load F_+ (and generates the force $-F_+$), and each minus-end directed motor feels the load $-F_-$ (and generates the force F_-).

Thus, the force experienced by a cargo being pulled by pulled by n_+ plus-end directed motors and n_- minus-end motors is given by

$$n_+F_+ = -n_-F_- = F_c \tag{2}$$

The sign of the force is taken as positive if a load was on the plus-end directed motors (i.e., if the force pointed into the minus-end direction).

The net unbinding rate for the plus-end directed motor is

$$n_+\epsilon_+ \exp[F_c/n_+F_{d+}] \tag{3}$$

where ε_+ indicates unloaded unbinding rate of single plus-end directed motor. The net rate for the binding of one plus-end directed motor is

$$(N_+ - n_+)\pi_+ \tag{4}$$

where π_+ is the binding rate of a single plus-end directed motor. The index "+" labels the plus-end directed motors properties and index "–" labels the minus-end directed motors properties.

The cargo force F_c is determined by the condition that the plus-end directed motors, which experience the force F_c/n_+, and the minus-end directed motors, which experience the force $-F_c/n_-$, move with the same velocity, which is the cargo velocity v_c:

$$v_c(n_+, n_-) = v_+(F_c/n_+) = -v_-(-F_c/n_-). \tag{5}$$

Here, the sign of the velocity is taken positive in the plus-end direction and negative in the minus-end direction.

In the case of stronger plus-end directed motors, $n_+F_{s+} > n_-F_{s-}$, the cargo force and velocity are given by the expressions

$$F_c = \left\{\frac{\frac{n_-F_{s-}}{v_{B-}}}{\frac{n_+F_{s+}}{v_{F+}} + \frac{n_-F_{s-}}{v_{B-}}}\right\}(n_+F_{s+}) + \left\{\frac{\frac{n_+F_{s+}}{v_{F+}}}{\frac{n_+F_{s+}}{v_{F+}} + \frac{n_-F_{s-}}{v_{B-}}}\right\}(n_-F_{s-}) \tag{6}$$

and

$$v_c(n_+, n_-) = \frac{n_+F_{s+} - n_-F_{s-}}{\frac{n_+F_{s+}}{v_{F+}} + \frac{n_-F_{s-}}{v_{B-}}} \tag{7}$$

In this case, the cargo moves to the plus-end direction with velocity $v_c > 0$.

In the opposite case of the stronger minus-end directed motors (i.e., $n_+F_{s+} < n_-F_{s-}$), in eqs. (6) and (7), the plus-end directed motor forward velocity v_{F+} has to be replaced by its backward velocity v_{B+}, and the minus motor backward velocity v_{B-} has to be replaced by its forward velocity v_{F-}. The cargo moves into the minus-end direction with velocity $v_c < 0$.

In case an external force F_{ext} is acting on the cargo, equation (2) can be written as

$$n_+F_+ = -n_-F_- + F_{ext} \tag{8}$$

and v_c can be written as

$$v_c(n_+, n_-) = \frac{n_+F_{s+} - n_-F_{s-} - F_{ext}}{\frac{n_+F_{s+}}{v_{F+}} + \frac{n_-F_{s-}}{v_{B-}}} \tag{9}$$

If cargo moves toward minus-end under an opposing force F_{ext} (which is then negative), the plus motor forward velocity v_{F+} should be replaced by its backward velocityv_{B+}, and the minus motor backward velocity v_{B-} by its forward velocity v_{F-}

Hence, in this case cargo velocity is given by

$$v_c(n_+, n_-) = \frac{n_+F_{s+} - n_-F_{s-} - F_{ext}}{\frac{n_+F_{s+}}{v_{B+}} + \frac{n_-F_{s-}}{v_{F-}}} \tag{10}$$

In the next section, we described our model geometry and how bidirectional tug-of-war models was extended to understand organelle positing by a team consisting of KIF5B, KIF1Bβ and dynein motors in a WT HeLa cell line.

Tug-of war model for lysosome transport

We model the cell (HeLa) as hemisphere of radius (R_{cell} = 16 μm) and the nucleus was also considered as a hemisphere of radius ($R_{nucleus}$ = 6 μm), as experiments were done with cells adhered to surface with average cell and nucleus diameters roughly ~ 32 μm and 12 μm, respectively (from microscopy images and measured using ImageJ). Microtubules were modelled as lines joining the nucleus to the cell periphery. We assumed that each cargo was transported along a separate microtubule and there was no steric hindrance among cargos while traveling on microtubules. Hence, assuming multiple cargos traveling on a single filament is not going to change our simulation results.

Due to the spherical symmetry of our model, we used spherical polar coordinates (r, θ, φ) to define the position of each cargo. The values of $\theta \in [0, \pi/2]$ and $\varphi \in [0, 2\pi]$ for each cargo were chosen randomly by Marsaglia's algorithm [2]. The initial radial position (r) of the cargo (central clustered, peripherally accumulated or normal steady-state distribution) was chosen according to the experiment to be modelled.

In our model, cargoes move radially along microtubules whose runs, pauses, reversals and detachments were simulated using bidirectional cargo transport model by Muller *et al.* [1].

Our WT HeLa cell line has only three different types of motors that move lysosomes [3]: kinesin-1 (KIF5B), kinesin-3 (KIF1Bβ) and dynein. Then, we had to extend the model from Muller

et. al. as follows: denoting kinesin-1 (KIF5B) with index $i = 1$, dynein with index $i = 2$, and kinesin-3 (KIF1Bβ) with index $i = 3$.

Hence, equation (1) is replaced by

$$v(F) = \begin{cases} v_F^i(1 - \frac{F}{F_s^i}), & F < F_s^i \\ v_B^i(1 - \frac{F}{F_s^i}), & F \geq F_s^i \end{cases} \qquad (i = 1, 2 \text{ and } 3) \tag{11}$$

where v_F^1, v_F^2, v_F^3 denote forward velocity, v_B^1, v_B^2, v_B^3 denote backward velocity, F_s^1, F_s^2, F_s^3 denotes stall force of of kinesin-1 (KIF5B), dynein and kinesin-3 (KIF1Bβ) motors, respectively.

Equation (2), which represents the force experienced by the cargo, is replaced by

$$(n^1 + n^3)F_+ = -n^2F_- = F_c \tag{12}$$

where n^1, n^2 and n^3 represent number of kinesin-1 (KIF5B), dynein and kinesin-3 (KIF1Bβ) motors pulling the cargo, respectively.

The net unbinding rate for motors is given by

$$r_u^i = (n^i \epsilon_o^i \exp\left(F_\pm / F_d^i\right) \tag{13}$$

where $\epsilon_o^1, \epsilon_o^2$ and ϵ_o^3 represent unbinding rates under zero load, F_d^1, F_d^2 and F_d^3 detachment forces for kinesin 1 (KIF5B), dynein and kinesin-3 (KIF1Bβ) motors, respectively.

Rate of binding of unbound of motors is given by

$$r_b^i = (N^i - n^i)\pi_o^i \tag{14}$$

where π_o^1, π_o^2 and π_o^3 represent binding rates, and N^1, N^2 and N^3 represent total number of kinesin-1 (KIF5B), dynein and kinesin-3 (KIF1Bβ) motors present bound to cargo surface, respectively.

Hence, in the case of stronger plus-directed motors, $n^1F_s^1 + n^3F_s^3 > n^2F_s^2$, the cargo force and velocity are given by the expressions

$$v_c(n^1, n^2, n^3) = \frac{n^1F_s^1 + n^3F_s^3 - n^2F_s^2}{\frac{n^1F_s^1}{v_F^1} + \frac{n^3F_s^3}{v_F^3} + \frac{n^2F_s^2}{v_B^2}} \tag{15}$$

and, force acting on the cargo is given by,

$$F_c = \left\{ \frac{\frac{n^2F_s^2}{v_B^2}}{\frac{n^1F_s^1}{v_F^1} + \frac{n^3F_s^3}{v_F^3} + \frac{n^2F_s^2}{v_B^2}} \right\} (n^1F_s^1 + n^3F_s^3) + \left\{ \frac{\frac{n^1F_s^1}{v_F^1} + \frac{n^3F_s^3}{v_F^3}}{\frac{n^1F_s^1}{v_F^1} + \frac{n^3F_s^3}{v_F^3} + \frac{n^2F_s^2}{v_B^2}} \right\} (n^2F_s^2) \quad (16)$$

If the plus end motors are weaker than the minus end motors i.e. $n^1F_s^1 + n^3F_s^3 < n^2F_s^2$ then the velocity of cargo is given by,

$$v_c(n^1, n^2, n^3) = \frac{n^1F_s^1 + n^3F_s^3 - n^2F_s^2}{\frac{n^1F_s^1}{v_B^1} + \frac{n^3F_s^3}{v_B^3} + \frac{n^2F_s^2}{v_F^2}} \quad (17)$$

and, force acting on the cargo is given by

$$F_c = \left\{ \frac{\frac{n^2F_s^2}{v_F^2}}{\frac{n^1F_s^1}{v_B^1} + \frac{n^3F_s^3}{v_B^3} + \frac{n^2F_s^2}{v_F^2}} \right\} (n^1F_s^1 + n^3F_s^3) + \left\{ \frac{\frac{n^1F_s^1}{v_B^1} + \frac{n^3F_s^3}{v_B^3}}{\frac{n^1F_s^1}{v_B^1} + \frac{n^3F_s^3}{v_B^3} + \frac{n^2F_s^2}{v_F^2}} \right\} (n^2F_s^2) \quad (18)$$

In our model, run lengths and run velocities, individual cargo trajectories were generated using the Gillespie algorithm as used by Muller *et al.* [1,4] for the motor attachment/detachment kinetics.

Simulations were performed to generate 500 cargo trajectories each trajectory starting from initial radial position (r) of the cargo (central clustered/peripherally accumulated/normal steady-state distribution) and randomly chosen angular position *i.e.* θ and φ. To model cargo release from the cell periphery (due to dissociation of mCh-KIF5B*-strep from lysosomes after biotin addition) we put r = R_{cell} for each cargo with $N^1 = 1$, $N^2 = 2$ and $N^3 = 8$ motors. To model cargo release from cell center (due to dissociation of strep-KIFC1*-mCh after biotin addition) we put r = $R_{nucleus}$ for each cargo with $N^1 = 1$, $N^2 = 2$ and $N^3 = 8$ motors. For control experiment, i.e. normal steady-state distribution, the radial positions of the cargoes were chosen randomly between the value of $R_{nucleus}$ and R_{cell} with $N^1 = 1$, $N^2 = 2$ $N^3 = 8$ motors.

In our simulations, cargo was allowed to move with the velocity v_c in the intervals between the attachment/detachment events. Simulations were performed until all motors were detached or a total simulation time of 30 minutes was reached. Parameters used in simulations are given in Supplementary Table 1.

Supplementary Table 1: Parameters used in simulation of model of organelle positioning

	Kinesin-1 (KIF5B)		Dynein		Kinesin-3 (KIF1Bβ)	
Parameter (unit)	Symbol	Value [Source]	Symbol	Value [Source]	Symbol	Value [Source]
Stall Force (pN)	F_s^1	2.5 [5]	F_s^2	2.5 [5]	F_s^3	0.15 [6]
Detachment Force (pN)	F_d^1	2.0 [5]	F_d^2	1.74 [5]	F_d^3	0.5 [7]
Binding rate (s^{-1})	π_o^1	1.0 (for $r < r_0$)[#]<br>0.0 (for $r > r_0$)[#]	π_o^2	5.0 [5]	π_o^3	6.0 [8,9]
Unbinding rate (s^{-1})	ϵ_o^1	1.0 [5]	ϵ_o^2	1.0 [5]	ϵ_o^3	1.43 [7]
Forward velocity (nm/s)	v_F^1	600	v_F^2	600	v_F^3	1350 [6]

[#]Kinesin-1 motors were found to be active near the nucleus only, by Guardia *et al.* [10]. Hence, binding rate of kinesin-1 set to zero for $r > r_0$. [$]Kinesin-3 motors were found active away from nucleus [10]. Soppina *et al.*[8] predicted that binding rate of kinesin-3 increases due to the presence of K loop which has not been measured experimentally. The binding rate of kinesin-3 is predicted to be between 0.1 s^{-1} to 10.0 s^{-1} by Nishinari *et al.*[9]. r_0 is the radial distance which was taken randomly between 8 μm and 10 μm in each microtubule in the cell.

References

[1] Müller, M. J., Klumpp, S., and Lipowsky, R. (2008). Tug-of-war as a cooperative mechanism for bidirectional cargo transport by molecular motors. *Proc Natl Acad Sci U S A.*, **105**, 4609-4614.

[2] Weisstein, Eric W. "Sphere Point Picking." From *MathWorld*--A Wolfram Web Resource. http://mathworld.wolfram.com/SpherePointPicking.html

[3] Pu, J., Guardia, C. M., Keren-Kaplan, T., and Bonifacino, J. S. (2016). Mechanisms and functions of lysosome positioning. *J Cell Sci.* **129**, 4329-4339.

[4] Gillespie, D. T. (1977). Exact stochastic simulation of coupled chemical reactions. *J Phys Chem.*, **81**, 2340-2361.

[5] Kunwar, A., Tripathy, S. K., Xu, J., Mattson, M. K., Anand, P., Sigua, R., Vershinin, M., McKenney, R. J., Clare, C. Y., Mogilner, A. and Gross, S. P., (2011). Mechanical stochastic tug-of-war models cannot explain bidirectional lipid-droplet transport. *Proc Natl Acad Sci U S A.*, **108**, 18960-18965.

[6] Siddiqui, N., and Straube, A. (2017). Intracellular cargo transport by kinesin-3 motors. *Biochemistry (Moscow)*, **82**, 803-815.

[7] Arpağ, G., Shastry, S., Hancock, W. O., and Tüzel, E. (2014). Transport by populations of fast and slow kinesins uncovers novel family-dependent motor characteristics important for in vivo function. *Biophys J.*, **107**, 1896-1904.

[8] Soppina, V., and Verhey, K. J. (2014). The family-specific K-loop influences the microtubule on-rate but not the superprocessivity of kinesin-3 motors. *Mol Biol Cell.*, **25**, 2161-2170.

[9] Nishinari, K., Okada, Y., Schadschneider, A., and Chowdhury, D. (2005). Intracellular transport of single-headed molecular motors KIF1A. *Phys Rev Lett.*, **95**, 118101.

[10] Guardia, C. M., Farías, G. G., Jia, R., Pu, J., and Bonifacino, J. S. (2016). BORC functions upstream of kinesins 1 and 3 to coordinate regional movement of lysosomes along different microtubule tracks. *Cell reports*, **17**, 1950-1961.
